# Osteochondroprogenitor cells and neutrophils expressing p21 and senescence markers modulate fracture repair

**DOI:** 10.1172/JCI179834

**Published:** 2024-05-16

**Authors:** Dominik Saul, Madison L. Doolittle, Jennifer L. Rowsey, Mitchell N. Froemming, Robyn L. Kosinsky, Stephanie J. Vos, Ming Ruan, Nathan K. LeBrasseur, Abhishek Chandra, Robert J. Pignolo, João F. Passos, Joshua N. Farr, David G. Monroe, Sundeep Khosla

**Affiliations:** 1 Division of Endocrinology and; 2 Robert and Arlene Kogod Center on Aging, Mayo Clinic, Rochester, Minnesota, USA.; 3 Department of Trauma and Reconstructive Surgery, BG Clinic, University of Tübingen, Tübingen, Germany.; 4 Division of Gastroenterology and Hepatology, Mayo Clinic, Rochester, Minnesota, USA.; 5 Robert Bosch Center for Tumor Diseases, Stuttgart, Germany.; 6 Department of Physical Medicine and Rehabilitation and; 7 Department of Physiology and Biomedical Engineering, Mayo Clinic, Rochester, Minnesota, USA.

**Keywords:** Aging, Bone biology, Bone disease, Cellular senescence

## Abstract

Cells expressing features of senescence, including upregulation of p21 and p16, appear transiently following tissue injury, yet the properties of these cells or how they contrast with age-induced senescent cells remains unclear. Here, we used skeletal injury as a model and identified the rapid appearance following fracture of p21^+^ cells expressing senescence markers, mainly as osteochondroprogenitors (OCHs) and neutrophils. Targeted genetic clearance of p21^+^ cells suppressed senescence-associated signatures within the fracture callus and accelerated fracture healing. By contrast, p21^+^ cell clearance did not alter bone loss due to aging; conversely, p16^+^ cell clearance, known to alleviate skeletal aging, did not affect fracture healing. Following fracture, p21^+^ neutrophils were enriched in signaling pathways known to induce paracrine stromal senescence, while p21^+^ OCHs were highly enriched in senescence-associated secretory phenotype factors known to impair bone formation. Further analysis revealed an injury-specific stem cell–like OCH subset that was p21^+^ and highly inflammatory, with a similar inflammatory mesenchymal population (fibro-adipogenic progenitors) evident following muscle injury. Thus, intercommunicating senescent-like neutrophils and mesenchymal progenitor cells were key regulators of tissue repair in bone and potentially across tissues. Moreover, our findings established contextual roles of p21^+^ versus p16^+^ senescent/senescent-like cells that may be leveraged for therapeutic opportunities.

## Abstract 2

Unique populations of senescent and senescent-like cells impair fracture repair.

## Graphical abstract

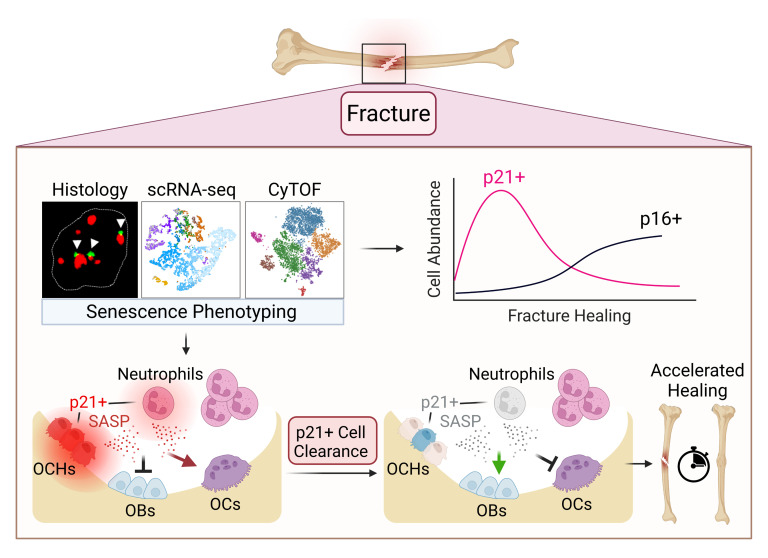

## Introduction

At the cellular level, aging is characterized by several hallmarks, including cellular senescence (1), which is driven by an increase in the cyclin-dependent kinase inhibitors *Cdkn1a* (p21) and/or *Cdkn*2a (p16) (2). Senescent cells also demonstrate a senescence-associated secretory phenotype (SASP), consisting of proinflammatory cytokines, multiple chemokines, and matrix-degrading proteins, driving tissue dysfunction in a paracrine and systemic manner (3). There is now considerable evidence that senescent cells accumulate with age across tissues and that clearance of these cells in mice using either genetic or pharmacologic approaches ameliorates multiple aging phenotypes, including frailty, osteoporosis, cardiovascular disease, metabolic dysfunction, and others (for a review, see ref. 4). This has led to intense interest in the development of compounds (senolytics) that target senescent cells to ameliorate these age-associated morbidities (5).

In addition to their role in aging, however, cells expressing features of senescence also appear rapidly following tissue injury and modulate the repair process (6). In contrast to age-associated senescent cells, these injury-related senescent-like cells remain relatively poorly characterized. Moreover, they appear to play variable roles in tissue repair, with evidence of a beneficial role in the healing of skin wounds (7) and lung repair following some (8), but not other forms (9), of lung injury versus a detrimental role in bone (10, 11) or muscle repair (12). In addition to understanding their fundamental biology, it is clearly important to characterize these injury-related senescent-like cells so we can better define the benefits versus risks of senolytic therapies for various age-associated morbidities as they rapidly move to the clinic.

Our group previously demonstrated (10) that cells expressing features of senescence — i.e., increased p16 or p21 expression, production of a SASP, and evidence of irreversible telomeric DNA damage (telomere-associated foci [TAFs], ref. 13; arguably one of the definitive assays for senescent cells) — accumulate transiently in the fracture callus. Pharmacologically targeting these cells by senolytic drugs (dasatinib + quercetin [D + Q]) accelerated both the time course and ultimate biomechanical strength of the healed fracture (10), as also confirmed by Liu et al. (11). In recent studies (14), we used cytometry by time-of-flight (CyTOF) as well as single-cell RNA sequencing (scRNA-seq) to rigorously define senescent cells in the context of aging at the single-cell level consistent with criteria outlined by the International Cell Senescence Association (15): upregulation of p16 and/or p21, growth arrest, upregulation of a SASP and antiapoptotic pathways, and evidence of DNA damage. In the present study, we used these validated tools to define the cellular identity and functional characteristics of cells expressing features of senescence following tissue injury using fracture as our model and contrasted these injury-related senescent-like cells to senescent cells associated with aging. We first characterized cells expressing senescence markers, including p21 and p16, in the fracture callus using CyTOF. Next, given the increasing evidence that p21 and p16 may be different functionally (16), we compared the effects of genetic clearance of p21^+^ versus p16^+^ cells on fracture healing using newly developed *p21-ATTAC* (17) as well as established *p16-INK-ATTAC* mouse models (18, 19). We further used CyTOF complemented by scRNA-seq analysis to provide a detailed characterization of the cells modulating fracture healing. Finally, we compared our findings following bone injury to available data in the context of muscle injury and identified very analogous mesenchymal progenitor populations in muscle that develop features of injury-related senescence, indicating that our findings likely extend across tissues. Collectively, our studies identify mesenchymal progenitor and immune populations expressing features of senescence following tissue injury that differ in important ways from classical senescent cells associated with aging, but which could nonetheless be targeted to enhance fracture healing and potentially facilitate repair in other tissues.

## Results

### Appearance of p21^+^ and p16^+^ cells expressing features of senescence during fracture healing.

**Figure 1:**
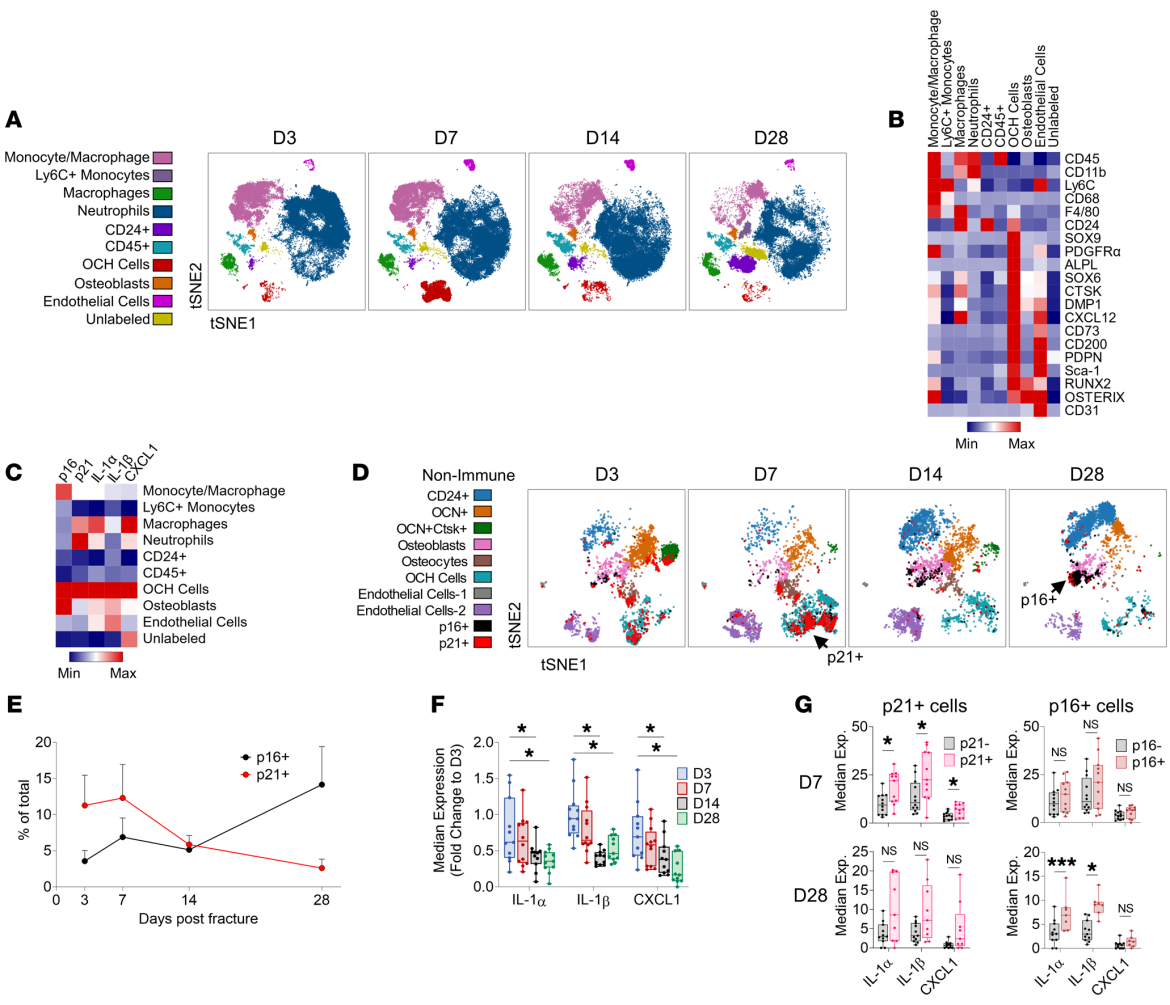
p21^+^ and p16^+^ cells appear in a divergent manner during fracture healing. (**A**) *t*-SNE visualization of clustered cell populations across murine fracture healing by CyTOF. (**B**) Heatmap representation of identification and (**C**) senescence and SASP marker median expression across all clusters. (**D**) *t*-SNE visualization of nonimmune (CD45^–^CD11b^–^) callus cells across fracture healing, overlaid with p16^+^ (black) and p21^+^ cells (red). (**E**) p16^+^ and p21^+^ cell abundances across fracture healing in nonimmune cells. (**F**) SASP marker median expression throughout fracture healing in all nonimmune cells and (**G**) in p16^+^ and p21^+^ nonimmune cells. Day 3: *n* = 12 mice (6 female, 6 male); day 7: *n* = 12 mice (6 female, 6 male); day 14: *n* = 12 mice (6 female, 6 male); day 28: *n* = 11 mice (6 female, 5 male). *P < 0.05; ***P < 0.001 by 1-way ANOVA with Tukey’s correction for multiple comparisons (**F**) or Mann-Whitney *U* test (**G**).

To investigate potential injury-related senescent-like cells that arise during fracture healing, we performed CyTOF on single-cell suspensions from digested callus samples throughout the fracture healing process using antibodies, including those for p16 and p21 that we have previously extensively validated (14). To capture the 4 stages of fracture healing (inflammatory, soft callus, hard callus, and remodeling phase), we performed a diaphyseal tibial fracture in 47 C57BL/6 mice (age 4 months) and harvested the newly formed callus on days 3, 7, 14, and 28. The minced and digested callus cells were analyzed by CyTOF (Figure 1A and Supplemental Figure 1A; supplemental material available online with this article; https://doi.org/10.1172/JCI179834DS1). We identified both mesenchymal and immune cell populations with unique abundance patterns throughout fracture healing (Figure 1, A and B). p16 and p21, previously observed to be expressed in the fracture callus (10, 11), were expressed in both mesenchymal and immune cell populations, with osteochondroprogenitor (OCH) cells, osteoblasts, and monocytes/macrophages expressing high levels of p16, and OCH cells and neutrophils expressing high levels of p21 (Figure 1C and Supplemental Figure 1B). Importantly, we found that coexpression of SASP factors with p16 and p21 was particularly strong in OCH cells (Figure 1C), which highly expressed osteogenic (Runx2, Osterix, ALPL, and DMP1), chondrogenic (Sox9 and Sox6), and progenitor (CD200, PDGFRα, and CD73) markers (Figure 1B).

To investigate the mesenchymal progenitor cells at higher resolution, nonimmune (CD45^–^CD11b^–^) callus cells were reclustered and phenotyped for their senescence profile (Figure 1D and Supplemental Figure 1, C–E). p21^+^ cells appeared early in fracture healing (days 3–7), predominantly as OCH cells, while p16^+^ cells appeared late (days 14–28) as osteoblasts (Figure 1D). This temporal expression pattern for p16 and p21 was observed among all nonimmune cells (Figure 1E). Notably, the existence of p21^+^ cells coincided with high inflammation, demonstrated by an early peak (day 3) in expression of SASP proteins IL-1α, IL-1β, and CXCL1 (Figure 1F). Moreover, SASP markers were enriched in p21^+^ cells at an early stage (days 3–7), but not at a late stage (day 28), while the opposite was true for p16^+^ cells (Figure 1G).

### Genetic clearance of p21^+^, but not p16^+^, cells accelerates fracture healing.

**Figure 2:**
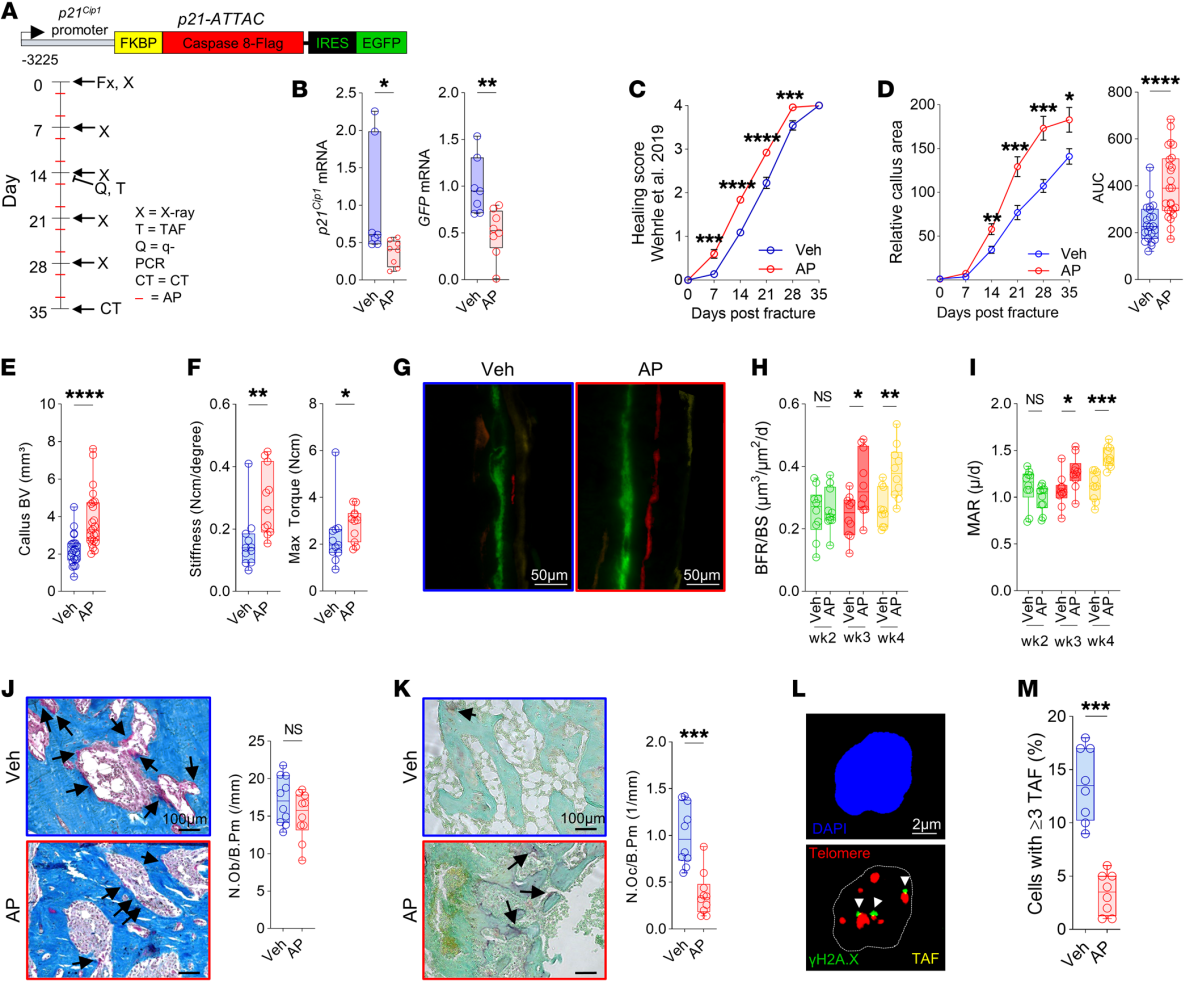
Clearance of p21^+^ cells accelerates fracture healing by increasing bone formation rates and reducing osteoclast numbers. (**A**) Schematic of the *p21-ATTAC* transgene and overall study design. *p21-ATTAC* mice (4–6 months old) were used to selectively clear p21^+^ cells through AP administration twice weekly over a 5-week fracture healing time course. (**B**) qRT-PCR measurement of *p21^Cip1^* and GFP (*p21-ATTAC* transgene) mRNA expression after AP treatment on day 14. (**C**) Fracture healing score (described by Wehrle et al.; ref. 21), and (**D**) callus area as measured by weekly x-rays. (**E**) μCT of callus bone volume (BV). (**F**) Tibial stiffness and maximal torque measured by biomechanical testing. (**G**–**I**) Histomorphometric analysis of bone formation rate per bone surface (BFR/BS) and mineral apposition rate (MAR) through weekly injections of bone-labeling dyes (see Methods). Scale bars: 50 μm. (**J**) Histological quantification of osteoblasts through Masson’s trichrome staining. (**K**) Histological quantification of osteoclasts through tartrate-resistant acid phosphatase (TRAP) staining. Scale bars: 100 μm (**J** and **K**). Arrows in **J** and **K** indicate osteoblasts and osteoclasts, respectively. (**L**) Telomere-associated foci (TAF) staining (day 14) for DNA damage. Scale bar: 2 μm. (**M**) Quantification of cells exhibiting 3 or more TAF per cell. *n* = 8–11 (**B** and **F**–**M**) or *n* = 22–25 (**C** and **D**) per treatment, equally split by sex. *P < 0.05; **P < 0.01; ***P < 0.001; ****P < 0.0001 by Mann-Whitney *U* test (**B**, **D**, **E**, and **J**–**M**), 2-way ANOVA with Šidák’s correction (**C**, **D**, and **F**), or multiple *t* test with FDR correction (**H** and **I**).

To evaluate the functional contribution of inflammatory p21^+^ versus p16^+^ cells toward fracture healing, we first leveraged the *p21-ATTAC* mouse model (Figure 2A), recently validated by our laboratory (17). This *p21-ATTAC* mouse (analogous to the *p16-INK-ATTAC* mouse; ref. 18) contains a “suicide” transgene driven by the *p21^Cip1^* promoter (20), whereby administration of AP20187 (AP) induces caspase 8–driven apoptosis in p21^+^ cells. Figure 2A also shows the design of the study, including the time points of twice-weekly AP administration following fracture. Quantitative real-time polymerase chain reaction (qRT-PCR) analysis of the callus area demonstrated a marked reduction in both p21 mRNA (*Cdkn1a*) and the *p21-ATTAC* transgene (*eGFP*) following AP treatment (Figure 2B). In the 5-week healing course of a transverse tibial fracture, we found that the x-ray–based healing score (21) was consistently improved in mice cleared of p21^+^ cells (Figure 2C). We next performed in-depth callus size measurements on a weekly basis and found the relative callus area to be significantly increased in the AP-treated mice from the second week onward (Figure 2D). At the conclusion of fracture healing, callus bone volume (by μCT) as well as biomechanical stiffness and maximum torque were increased in mice cleared of p21^+^ cells (Figure 2, E and F). Using fluorescent labeling of the newly formed callus area (22), we found an increase in the bone formation rate/bone surface (BFR/BS) and mineral apposition rate (MAR) in weeks 3 and 4, with clearance of p21^+^ cells (Figure 2, G–I). Interestingly, we found no difference in osteoblast numbers between the treatment groups (Figure 2J), suggesting that the increases in MAR and BFR in AP-treated mice were due primarily to an increase in osteoblast activity, rather than number. In addition, the number of osteoclasts per bone perimeter were reduced in the AP-treated mice (Figure 2K).

We next evaluated whether clearance of p21^+^ cells was associated with a reduction in senescent cell signatures. To do so, we performed TAF analysis (Figure 2L), which identifies sites of irreversible telomeric DNA damage, a hallmark of senescent cells (13). We have previously demonstrated that TAF increase markedly in the fracture callus, peaking on day 14 following fracture and subsequently returning toward baseline levels (10). Here, we observed a marked reduction in TAF^+^ cells in the AP- versus vehicle-treated mice on day 14 (Figure 2M). Thus, clearance of p21^+^ cells effectively reduced hallmarks of senescence, resulting in accelerated fracture healing and stronger bone.

To evaluate whether the clearance of p16^+^ cells also altered the time course of fracture healing, we used the *p16-INK-ATTAC* model (Supplemental Figure 2A). We confirmed adequate functionality of the *p16-INK-ATTAC* model by demonstrating downregulation of the *Casp8* portion of the transgene cassette in the fracture callus following AP treatment, consistent with clearance of cells expressing the *p16*-driven suicide transgene (Supplemental Figure 2B). Note that the primers used for *Casp8* are specific for the human transcript encoded by the transgene (23). Weekly x-ray analyses showed a small, but insignificant, difference in the callus formation rate, with a marginal acceleration in radiographic callus formation in the AP-treated group in the late stage (day 28, Supplemental Figure 2C). However, the resulting total callus volume of the healed bones remained unchanged (Supplemental Figure 2D). Both our CyTOF analyses and previous studies (10) demonstrated that p16^+^ cells begin to emerge within the callus around day 14 after fracture. To detect the expected effect of AP on senescent cells, we performed TAF analyses for telomeric DNA damage, but did not find a significant difference in cells expressing this feature of senescence within the callus region with AP treatment (Supplemental Figure 2, E and F). These findings thus indicate that targeted clearance of p16^+^ cells does not significantly improve initial fracture healing, but may have a marginal effect at a later stage (e.g., day 28, reflected by a higher AUC under the fracture healing curve; Supplemental Figure 2C) when p16^+^ cells are more prevalent (Figure 1E).

### Clearance of p21^+^ cells suppresses OCH- and neutrophil-derived factors driving osteoclast recruitment and inhibition of bone formation.

**Figure 3:**
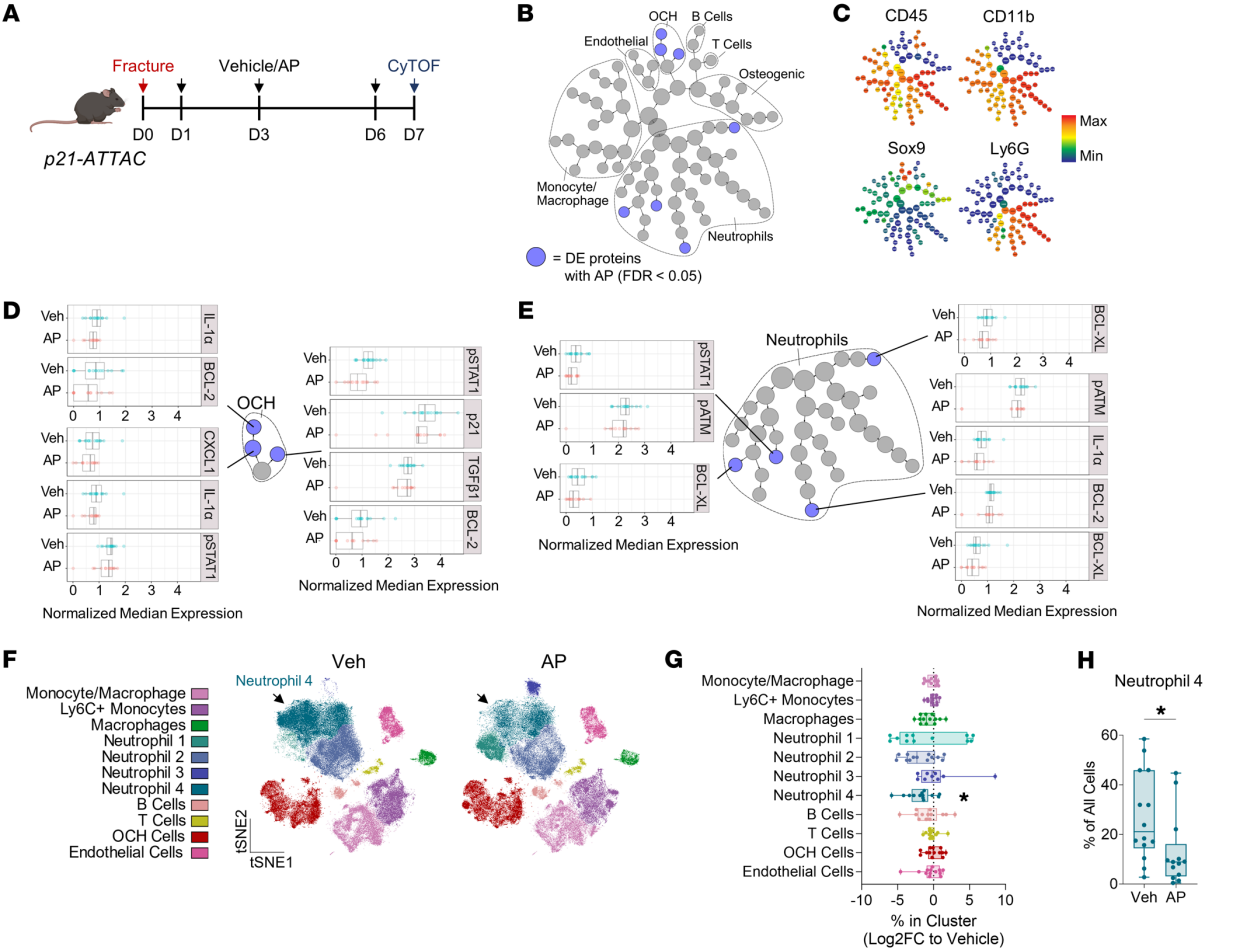
Clearance of p21^+^ cells suppresses factors driving osteoclast recruitment and inhibition of bone formation through targeting OCH cells and neutrophils. (**A**) Schematic outlining CyTOF analysis of callus cells after p21^+^ cell clearance in *p21-ATTAC* mice. (**B**) CITRUS analysis reveals reduced expression (blue dots; FDR < 0.05) of proteins in OCH and Neutrophil cell clusters. (**C**) CITRUS expression plots for key identification markers. (**D**) CITRUS results of differential expression between vehicle- and AP-treated groups in OCH (**D**) and Neutrophil (**E**) clusters; all FDR < 0.05. (**F**) *t*-SNE visualization and FlowSOM clustering of callus cells from *p21-ATTAC* mice treated with either vehicle (Veh) or AP. (**G**) Quantification of changes in cell cluster percentages after AP treatment (log_2_[fold change]; 2-way ANOVA with Šidák’s multiple-comparison test). (**H**) Quantification of absolute changes in the Neutrophil-4 cluster after AP treatment (Mann-Whitney *U* test). *n* = 16 vehicle-treated mice (8 female, 8 male), *n* = 13 AP-treated mice (7 female, 6 male). *P < 0.05.

To investigate how clearance of p21^+^ cells enhanced fracture healing, we performed single-cell CyTOF phenotyping of callus cells from *p21-ATTAC* mice following clearance of p21^+^ cells using AP (Figure 3A). Using CITRUS analysis, which generates separately stratified clusters from the original data set to observe statistical differences (24), we found that AP administration led to downregulation of markers for the SASP, DNA damage, and antiapoptosis proteins specifically within OCH and neutrophil clusters (Figure 3, B–E, and Supplemental Figure 3A; note that all changes in Figure 3, D and E are statistically significant, with FDR < 0.05). Within the OCH cells, CXCL1, which is a potent chemoattractant for neutrophils (25) and osteoclasts (26), as well as TGF-β1, an inhibitor of mineralization and bone formation (11, 27), were significantly reduced by AP treatment (Figure 3D). Other signals suppressed in both OCH cells and neutrophils with AP treatment included IL-1α, known to stimulate osteoclastogenesis (28) and osteoblast apoptosis (29), and STAT1 signaling (measured by p-STAT1 expression), which inhibits fracture-related bone formation (30).

FlowSOM clustering of these cells (Supplemental Figure 3, B and C) demonstrated a significant reduction in a subset of neutrophils (Neutrophil-4) of over 50% after AP treatment (Figure 3, F–H) (vehicle: 27% of total cells vs. AP: 13%; *P* = 0.0125), but no changes in the abundance of OCHs or any other cell populations (e.g., macrophages, B, or T cells). In summary, we found that clearance of p21^+^ cells in the *p21-ATTAC* mice reduced the numbers of a specific subset of neutrophils, did not reduce numbers of inflammatory OCH cells, but did reduce the SASP of the inflammatory OCH cells, including factors related to osteoclast recruitment and suppression of bone formation.

### scRNA-seq analysis of inflammatory p21^+^ callus cells.

**Figure 4:**
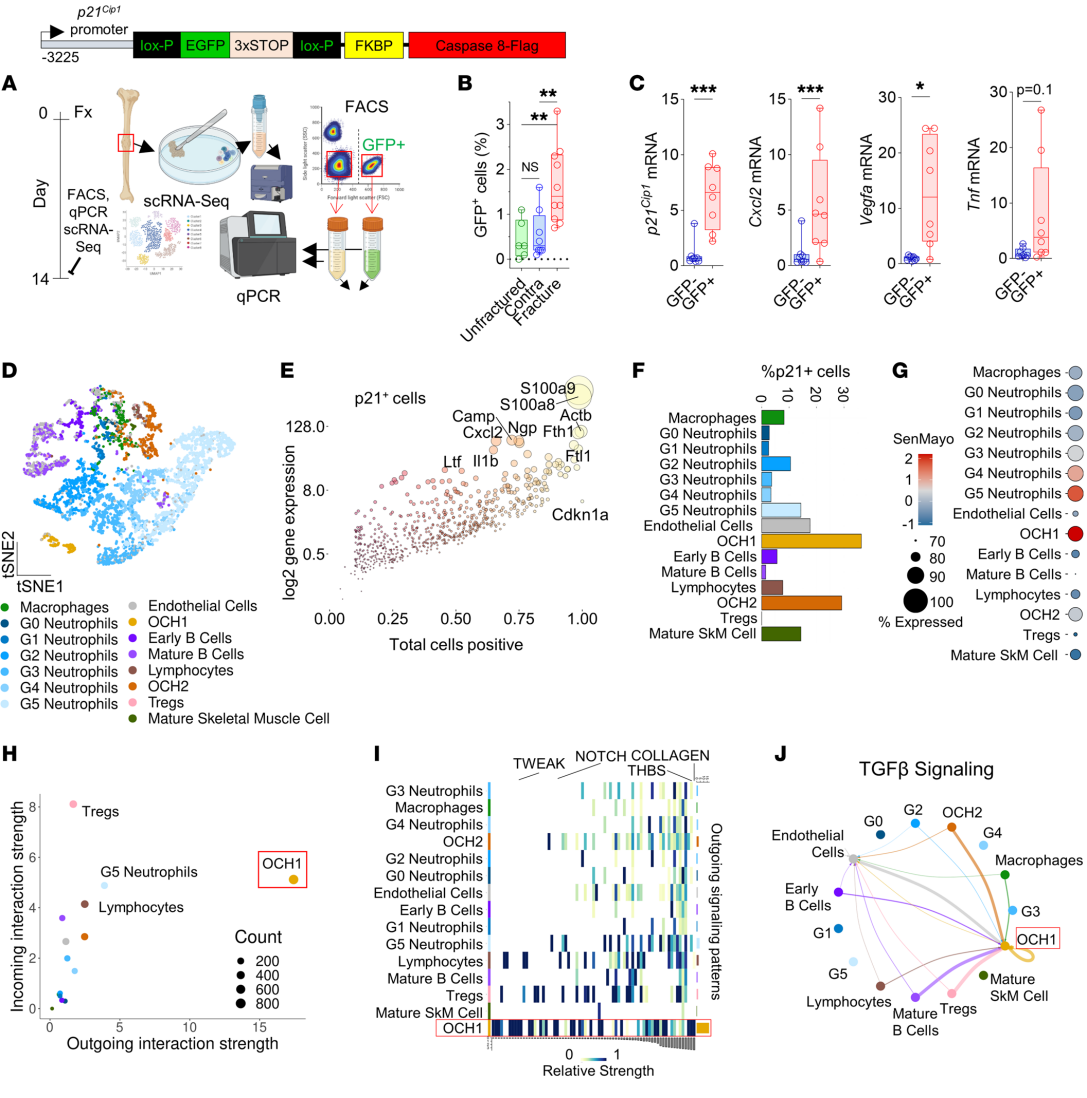
p21^+^ callus cells are largely highly secretory OCH cells and mature neutrophils. (**A**) Schematic of p21^+^ cell isolation and scRNA-seq using the p21 reporter mice. (**B**) GFP^+^ cells were significantly higher in the fractured compared with the unfractured contralateral side and an unfractured mouse tibia. *n* = 10 fractured (*n* = 4 female, *n* = 6 male), *n* = 8 contralateral sides (*n* = 4 female, *n* = 4 male), *n* = 6 unfractured (*n* = 3 female, *n* = 3 male). (**C**) *p21^Cip1^*, *Cxcl2*, *Vegfa*, and *Tnfa* mRNA expression was significantly enriched in GFP^+^ cells. *n* = 8 mice (*n* = 4 male, *n* = 4 female). (**D**) scRNA-seq analysis was performed on 5,994 total callus cells from *n* = 4 mice (*n* = 2 male, *n* = 2 female). (**E**) Differentially upregulated mRNA transcripts in p21^+^ cells. (**F**) Proportion of p21^+^ cells and (**G**) SenMayo gene enrichment analysis among clustered cell populations. (**H** and **I**) Predicted secretory strength relationships in callus cells among all signaling pathways and (**J**) TGF-β signaling by CellChat. *P < 0.05; **P < 0.01; ***P < 0.001 by 1-way ANOVA with Tukey’s correction (**B**) or Mann-Whitney *U* test (**C**, GFP^+^ vs. GFP^–^).

Given the critical role of p21^+^ cells in modulating fracture repair, we next used scRNA-seq to further investigate the inflammatory profile of these cells. To enrich for p21^+^ cells, we used a reporter mouse in which a validated fragment of the p21 promoter (17) was placed upstream of GFP (Figure 4A). This allowed for FACS isolation of p21^+^ (GFP^+^) and p21^–^ (GFP^–^) callus cells at 14 days after fracture (Figure 4A). We first validated the increase in GFP^+^ cells within the fracture site using qRT-PCR and found a significant enrichment of the GFP signal within the callus (Figure 4B). We also performed further qRT-PCR analysis of the GFP^+^ versus GFP^–^ cells and detected an increase in SASP markers (*Cxcl2*, *Vegfa*, and *Tnfa*) in the GFP^+^ population (Figure 4C). We then performed scRNA-seq followed by unbiased clustering, leading to 15 distinct clusters (Figure 4D). A number of SASP markers were enriched in p21^+^ cells, including *Cxcl2*, *Ccl4*, *Il1b*, as well as *Tgfb1* (Figure 4E) that has recently been shown to impair fracture healing (11). Consistent with our CyTOF data, the majority of p21^+^ cells were found within OCH cells (Figure 4F). Due to an increase in clustering variables compared with CyTOF, the scRNA-seq clustering further subgrouped the OCH cells into 2 populations (OCH1 and OCH2), with the OCH1 cells being perhaps earlier in the differentiation lineage (Supplemental Figure 4, A and B). Notably, the OCH1 cells also had the highest SASP profile (Figure 4G), as demonstrated by SenMayo gene set enrichment (31). Moreover, the OCH1 cells were predicted to have the highest outgoing strength of all signaling pathways among callus cell types (Figure 4, H and I), consistent with a highly secretory profile, particularly in TGF-β signaling (Figure 4J). In addition, OCH1 cells highly expressed activin A (encoded by *Inhba*) (Supplemental Figure 4C), a TGF-β superfamily member recently found to mark a distinct proliferative progenitor cell population in the fracture callus (32).

We also clustered the neutrophils into G1–G5 based on a previously published mouse neutrophil atlas (Figure 4D) (33). The G5 neutrophils had the highest percentage of p21^+^ cells among the 6 neutrophil populations (Figure 4F), had the second-highest outgoing signal interaction strength among all cell types (Figure 4H), were Ki67^–^ (Supplemental Figure 4D), and were the neutrophil population with the highest SenMayo and ROS scores (Supplemental Figure 4, E and F). G5 neutrophils are a mature neutrophil population predicted to arise from the peripheral blood (33). Note that due to the greater resolution of the scRNA-seq analysis as compared with CyTOF, we cannot unequivocally equate the G5 population with one of the 4 neutrophil populations identified above by CyTOF. However, like G5 neutrophils, the Neutrophil-4 population that was cleared with AP treatment contained the largest percentage of p21^+^ cells among all neutrophil subpopulations (Supplemental Figure 4, G and H). This suggests that the Neutrophil-4 population identified by CyTOF contains, or is highly enriched for, the G5 neutrophils found by scRNA-seq. This inflammatory neutrophil population was predicted to utilize the thrombospondin1 (THBS1)/CD47 pathway for communication with the OCH1 population (Supplemental Figure 4, I and J). This suggests G5 neutrophil secretion of THBS1, which binds to the CD47 receptor on the OCH1 population, an interaction known to induce paracrine senescence in mesenchymal cells (34, 35).

Next, we aimed to identify regulatory units for the OCH cells. Using SCENIC (36), we reconstructed a regulon network and, based on the AUC of a regulon activity heatmap, we identified Krüppel-like factor 4 (*Klf4*) to be the most prominent transcription factor for the OCH cells (Supplemental Figure 5, A and B). Importantly, *Klf4* has been shown to increase in response to inflammatory stimuli and mediate proinflammatory signaling and was of particular importance in the OCH1 and OCH2 clusters (Supplemental Figure 5C) (37).

### Evidence for the appearance of similar injury-related senescent-like cells following muscle injury.

To evaluate whether similar, inflammatory p21^+^ mesenchymal cell populations may be present not only following skeletal fracture, but also following injury across different tissues, we analyzed publically available scRNA-seq data following muscle injury (12). A mesenchymal progenitor cell population in muscle highly analogous to the OCH cells in bone are fibro-adipogenic progenitors (FAPs) (38). Interestingly, following muscle injury, FAPs not only expressed p21 (Supplemental Figure 6, A and B), they also demonstrated the highest expression of SASP-associated genes (Supplemental Figure 6C). Similar to the OCH1 cells, FAPS had the highest predicted outgoing signaling strength (Supplemental Figure 6, D and E), accompanied by strong TGF-β signaling to surrounding cells (Supplemental Figure 6F), including activin A expression (Supplemental Figure 6G). Of note, the neutrophil population, which was relatively small in this data set and thus could not be further subdivided, nonetheless also showed a communication pattern similar to the G5 population toward the OCH1 cells, consisting of the crucial THBS1/CD47 axis from neutrophils to FAPs (Supplemental Figure 6, H and I).

### p21^+^ OCHs are non–growth-arrested inflammatory callus cells.

**Figure 5:**
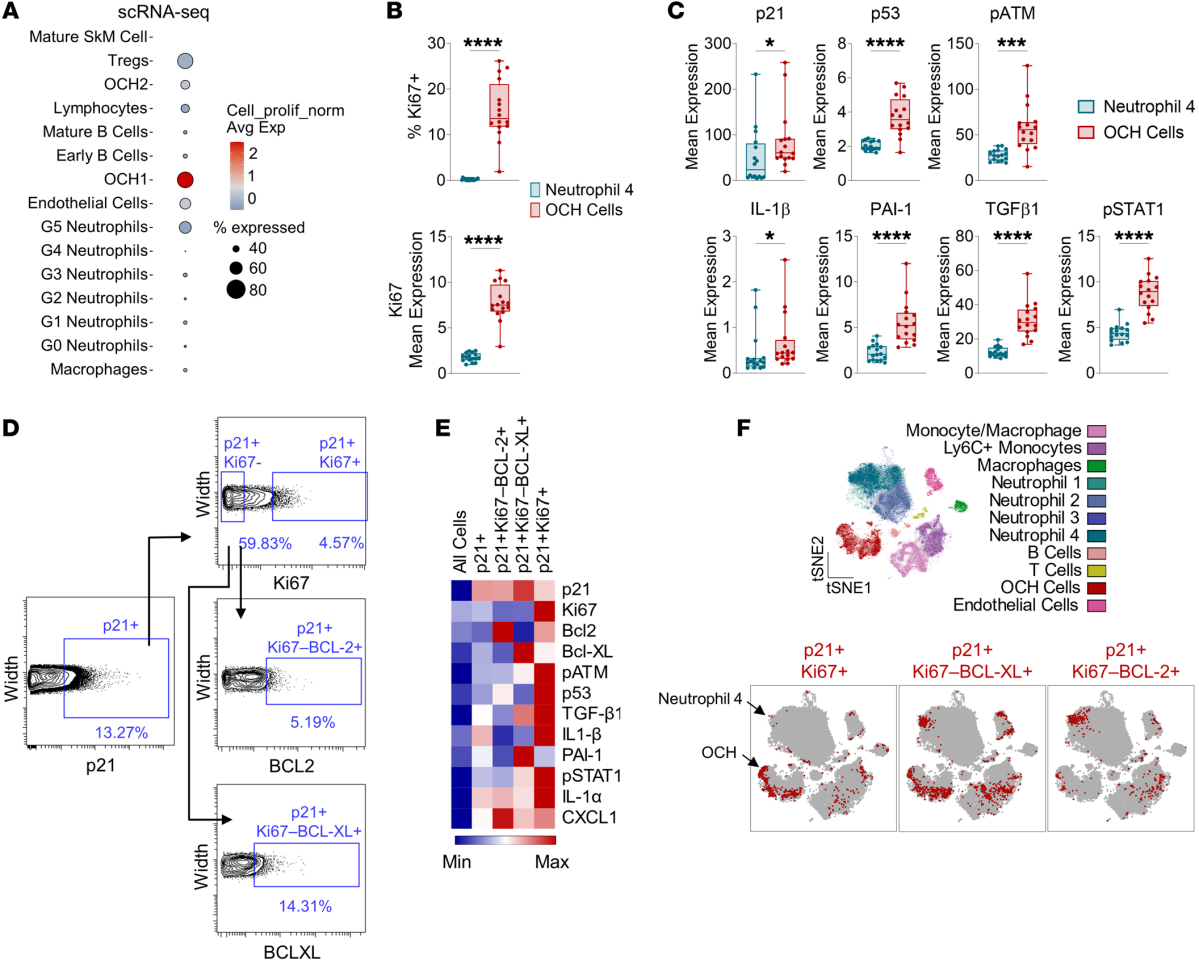
OCH cells are a proliferative population with a senescent-like phenotype. (**A**) Expression of cell proliferation gene set (GO_0008283) among callus cell populations identified by scRNA-seq (see Figure 4). *n* = 4 mice. (**B**) Percentage Ki67^+^ and Ki67 mean expression between OCH cell and Neutrophil-4 callus cell clusters identified by CyTOF (see Figure 3). (**C**) Senescence-related and SASP protein expression between OCH cells and Neutrophil-4 clusters. (**D**) Gating strategy for p21^+^/Ki67^+^, p21^+^Ki67^–^BCL2^+^, and p21^+^Ki67^–^BCL-XL^+^ cell populations in fractured mice. (**E**) Heatmap demonstrating mean expression of senescence-associated proteins in p21^+^ subsets by CyTOF. (**F**) *t*-SNE visualization of FlowSOM-clustered callus cells overlaid with p21^+^Ki67^+^, p21^+^Ki67^–^BCL-XL^+^, and p21^+^Ki67^–^BCL2^+^ cells (red). *n* = 16 mice (**B**–**F**). *P < 0.05, ***P < 0.001, ****P < 0.0001 by unpaired, 2-tailed *t* test (**B** and **C**: p53, p-ATM, PAI-1, TGF-β1, p-STAT1) or Mann-Whitney *U* test (**C**: p21, IL-1β).

OCHs exhibited the predominant senescence signature of all callus cells, including the highest percentage of p21^+^ cells and the highest expression of the SenMayo panel, yet it remained unclear why these cells were not reduced after targeted clearance in the *p21-ATTAC* mice. Surprisingly, although OCH1 cells demonstrated a clear senescent-like phenotype by scRNA-seq, they were found to have a high enrichment for proliferation-associated gene expression (Figure 5A). Moreover, out of the two p21^+^ cell populations shown to be affected by p21^+^ cell clearance by CyTOF, OCH cells were substantially higher in percentage of Ki67^+^ cells and Ki67 mean expression as compared with the Neutrophil-4 cells (Figure 5B). Paradoxically, OCH cells still demonstrated an otherwise clear senescent phenotype, demonstrating higher expression of senescence (p21 and p53), DNA damage (p-ATM), and detrimental SASP (IL-1β, PAI-1, TGF-β1, and p-STAT1) markers than Neutrophil-4 cells (Figure 5C).

We have previously found that, in the context of aging, skeletal cells marked by the senescence-associated cell cycle protein p21 or p16 contain both senescent and nonsenescent (i.e., non–growth-arrested) cell types, with senescent subsets defined as Ki67^–^ and positive for apoptosis-resistance proteins (14). In our callus cells, we subdivided p21^+^ cells by proliferative (Ki67^+^) versus nonproliferative (Ki67^–^) cells, and then further divided Ki67^–^ cells into subsets positive for senescence-associated apoptosis-resistance protein BCL-XL or BCL2 (Figure 5D). While we found that each population demonstrated an enriched SASP, the p21^+^Ki67^+^ subpopulation surprisingly exhibited the highest levels of a majority of senescence-associated markers in our CyTOF panel (Figure 5E). These p21^+^Ki67^+^ cells were highly enriched in OCH cells (Figure 5F). By contrast, the p21^+^Ki67^–^BCL2^+^ cells appeared predominantly within Neutrophil-4 cells (Figure 5F), while p21^+^Ki67^–^BCL-XL^+^ cells appeared in both Neutrophil-4 and OCH cell clusters (Figure 5F). Thus, the predominant inflammatory mesenchymal p21^+^ cell population in fracture healing is a previously uncharacterized p21^+^Ki67^+^ OCH population, rather than truly senescent, growth-arrested cells. However, the appearance of a neutrophil subpopulation expressing features of senescence (upregulation of p21, growth arrest, upregulation of BCL2 and/or BCL-XL) was unexpected, although perhaps not unprecedented, as a similar neutrophil population expressing features of senescence has recently been described in the setting of prostate cancer (39).

### The p21^+^ inflammatory subpopulation of OCHs demonstrates a skeletal stem cell expression profile and is injury specific.

**Figure 6:**
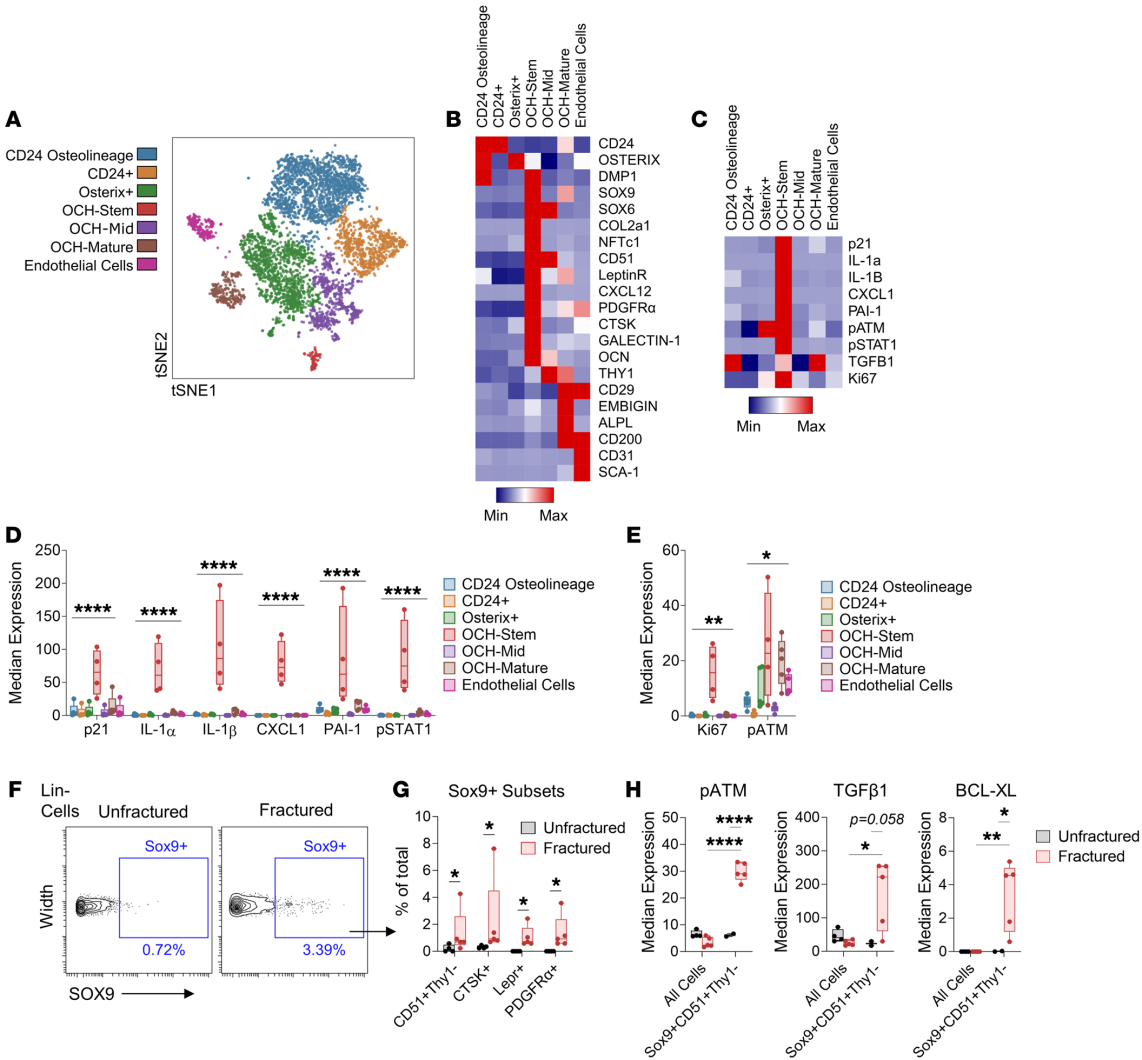
OCHs expressing SSC markers define an injury-specific senescent-like population. (**A**) *t*-SNE visualization of FlowSOM-clustered callus cell populations collected from fractured WT C57BL/6 mice. (**B** and **C**) Heatmap representation of mean protein expression of (**B**) identity and (**C**) senescence-associated markers. (**D** and **E**) Quantification of senescence-associated proteins among all clustered cell populations. (**F**) Gating strategy for Lin^–^Sox9^+^ cells in CyTOF samples isolated from either unfractured or fractured bones from young (4- to 6-month-old) mice. (**G**) Quantification of manually gated OCH-Stem clusters in both unfractured and fractured samples. (**H**) Quantification of senescence-associated proteins in manually gated OCH-Stem (Lin^–^Sox9^+^CD51^+^) cell populations compared to all cells in both unfractured and fractured samples. *n* = 5 fractured, *n* = 4 unfractured, all female. *P < 0.05; **P < 0.01; ****P < 0.0001 by 2-way ANOVA with Tukey’s multiple-comparison test (**D**, **E**, and **H**) or Mann-Whitney *U* test (**G**).

To investigate the OCH population further, we performed additional CyTOF analyses using an expanded antibody panel to include markers known to label skeletal stem cells (SSCs) (CD51, Ctsk, and LeptinR; refs. 40–42), chondrogenic cells (Col2a1 and NFATc1; refs. 43, 44), and non-stem stromal cells (Thy1 and Embigin; refs. 45, 46). In isolated callus cells, we found 3 separate OCH clusters (positive for Sox9 or Sox6) that expressed markers belonging to distinct stages of differentiation: OCH-Stem (CD51^+^LeptinR^+^PDGFRα^+^Ctsk^+^Thy1^–^), OCH-Mid (CD51^+^Thy1^+^), and OCH-Mature (CD29^+^CD200^+^Embigin^+^ALPL^+^) (Figure 6, A and B). Interestingly, the majority of senescence markers — including those found to be expressed in OCH cells in the previous analyses described above — were most enriched in OCH-Stem cells (Figure 6, C and D). Moreover, these cells were overall highest in Ki67 expression, and among the highest in p-ATM expression, suggesting a DNA-damaged, yet proliferative phenotype (Figure 6E). This OCH-Stem population appeared to be injury specific, as various manually gated Sox9^+^ subsets expressing OCH-Stem SSC markers were highly upregulated in the fracture callus, yet existed at extremely low or nonexistent levels in unfractured control mice (Figure 6, F and G). This injury-specific OCH-Stem population also demonstrated clear upregulation of p-ATM, TGF-β1, and BCL-XL compared with non-injured controls (Figure 6H). Overall, these data define an injury-related senescent-like OCH population expressing SSC markers that are expanded upon bone fracture and develop features of cellular senescence solely within the context of skeletal injury.

### Clearance of p21^+^ cells has no greater effect on fracture healing in aged mice and does not alleviate age-related bone loss.

**Figure 7:**
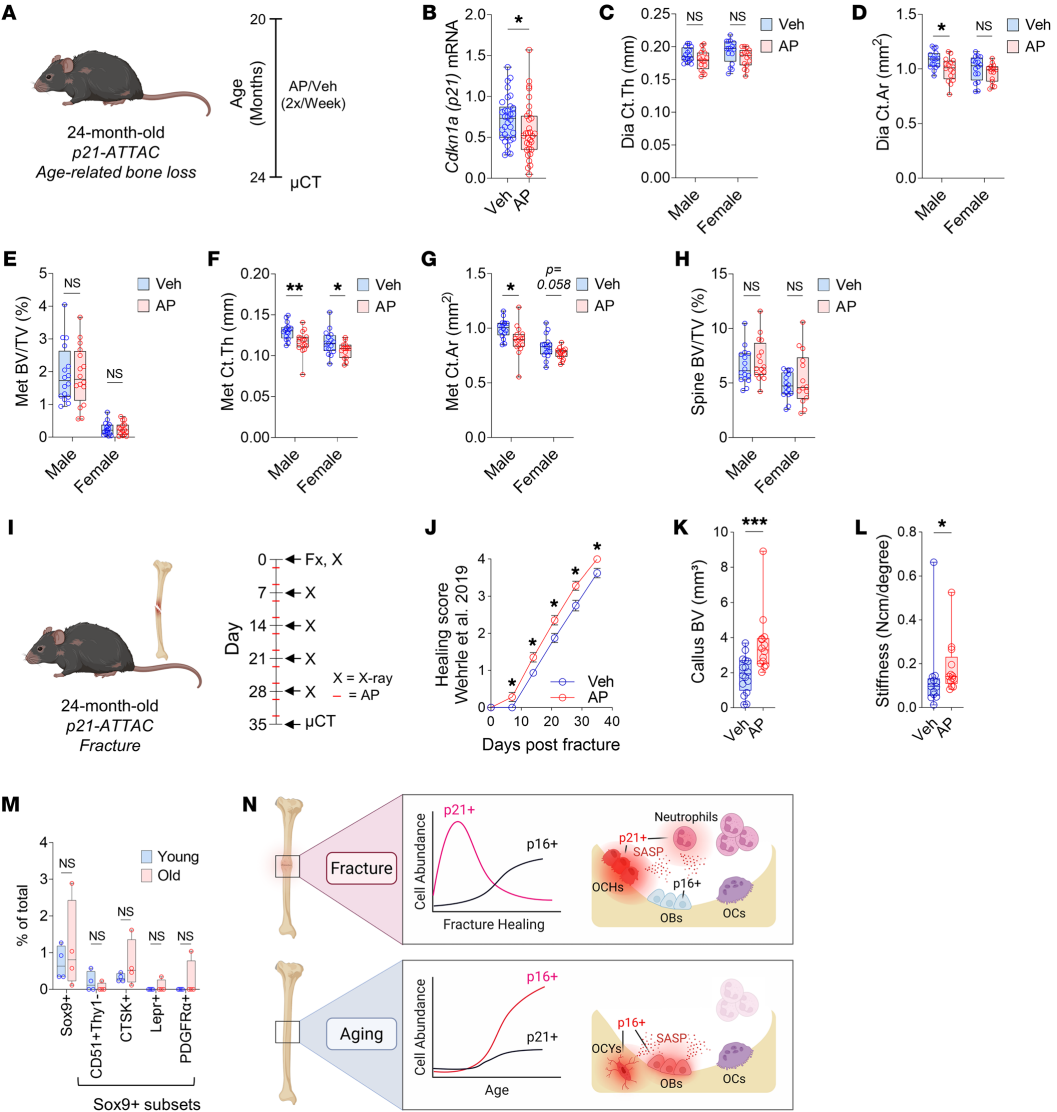
Detrimental effects of p21^+^ cells on bone metabolism are aging independent. (**A**) p21^+^ cell clearance was performed in old *p21-ATTAC* mice treated at 20 months of age for 4 months with either vehicle (Veh) (*n* = 32 mice: 16 male, 16 female) or AP (*n* = 32 mice: 16 male, 15 female) twice weekly until sacrifice at 24 months. (**B**) qRT-PCR measurement of *p21^Cip1^* mRNA expression. (**C**–**G**) Skeletal parameters measured at the femur by μCT: (**C**) diaphyseal (Dia) cortical thickness (Ct.Th), (**D**) Dia cortical area (Ct.Ar), (**E**) metaphyseal (Met) trabecular bone volume per total volume (BV/TV), (**F**) Met Ct.Th, and (**G**) Met Ct.Ar. (**H**) Trabecular BV/TV measured at the L5 lumbar vertebra. (**I**) Schematic for clearance of p21^+^ cells in old mice undergoing fracture repair; 24-month-old *p21-ATTAC* mice were used to selectively clear p21^+^ cells through treatment with either vehicle (*n* = 16 mice; 8 male, 8 female) or AP (*n* = 14 mice; 7 male, 7 female) twice weekly over a 5-week fracture healing time course. (**J**) Fracture healing score measured by weekly x-ray. (**K**) μCT of callus bone volume. (**L**) Tibial stiffness measured by biomechanical testing (*n* = 13 Veh: 6 male, 7 female. *n* = 13 AP: 7 male, 6 female). (**M**) CyTOF analysis of OCH-Stem population abundances among CD45^–^Lin^–^ nonimmune cells isolated from the digested hind limbs of young (6-month-old) and old (24-month-old) WT C57BL/6 mice (*n* = 4 mice per group, all female). (**N**) Schematic of results from injured young bone versus intact aging bone. OCHs, osteochondroprogenitors; OBs, osteoblasts; OCs, osteoclasts; OCYs, osteocytes. Note that this figure is a schematic and only provides a depiction of the cell populations rather than quantitative data. *P < 0.05; **P < 0.01; ***P < 0.001 by Mann-Whitney *U* test (**B** and **K**–**M**), unpaired, 2-tailed *t* test (**C**–**H**), or 2-way ANOVA with Šidák’s correction (**J**).

As senescence is clearly linked to aging, we next evaluated whether these inflammatory p21^+^ cells appear in the setting of aging and contribute to age-related bone loss. *p21-ATTAC* mice were thus aged to 20 months and treated with either vehicle or AP for 4 months to assess skeletal phenotypes at 24 months of age (Figure 7A), a treatment regimen previously shown to alleviate age-related bone loss in *p16-INK-ATTAC* mice (19). Upon reduction of p21^+^ cells, indicated by lower *Cdkn1a* mRNA expression in bone (Figure 7B), there were no beneficial changes in any skeletal parameter at any of the 3 sites examined (femur diaphysis, metaphysis, or lumbar spine) (Figure 7, C–H). The few statistically significant changes identified were, in fact, detrimental effects on femoral skeletal parameters (Figure 7, D, F, and G).

To test whether aging perhaps exacerbates the effect of p21^+^ cells on impairing fracture healing, we fractured 24-month-old *p21-ATTAC* mice and treated them with vehicle or AP for 5 weeks after fracture (Figure 7I), identical to our experiments in young (4-month-old) mice (Figure 2). Although there was still a beneficial effect of p21^+^ cell clearance on fracture healing, neither the acceleration (x-ray healing score) nor the endpoint effect (callus bone volume) was any greater in the aged versus the young mice (Figure 7, J–L). Using CyTOF on bone samples from 24-month-old mice, we found that there was no age-related increase in any of the OCH-Stem subpopulations found to express features of senescence in the setting of fracture (Figure 7M). This suggests that the detrimental effects of senescent-like p21^+^ cells on bone metabolism are independent of aging. In summary, clearance of p21^+^ cells in aged mice had no greater effect on fracture healing than in young mice and, in contrast to clearance of p16^+^ cells (19, 47), did not alleviate age-related bone loss.

## Discussion

In the present study, we identified and defined the functional role of cells expressing features of senescence during tissue repair, using fracture healing as our model. These injury-related senescent-like cells, which consisted principally of p21-expressing OCH cells and neutrophils, have similarities as well as some key differences from classical senescent cells associated with aging. Specifically, in the context of aging, these cells appear to be principally, if not exclusively, of mesenchymal origin (14, 48). Indeed, cellular senescence was originally defined by Hayflick and Moorhead for mesenchymal cells (49), and whether immune cells express the full features of classical senescent cells remains unclear. However, following fracture, there clearly was a subpopulation of neutrophils (Neutrophil-4 by CyTOF, G5 neutrophils by scRNA-seq) that expressed p21, were growth arrested, had evidence of DNA damage, expressed a SASP, and upregulated BCL2 and/or BCL-XL. A second population of these injury-related senescent-like cells consisted of mesenchymal OCH cells (OCH-Stem) that expressed p21, DNA damage markers, and a SASP, but also expressed proliferation markers (p21^+^Ki67^+^ cells by CyTOF, p21^+^ cells positive also for proliferation genes by scRNA-seq), in contrast to the growth-arrested classical senescent cells we previously identified in the context of skeletal aging (14). In addition, while classical senescent cells associated with aging are characterized by their persistence over time, the injury-related senescent-like cells were relatively transient, as our previous studies demonstrated that the TAF^+^ cells in the fracture callus had largely disappeared by day 28 following fracture (10). Finally, although our studies focused on skeletal injury, our demonstration of p21-expressing mesenchymal FAPs following muscle injury with features very analogous to the OCH cells suggests that these injury-related mesenchymal progenitor senescent-like cells appear following not just fracture, but following injury across tissues.

Our data further demonstrated that clearance of p21^+^ cells using a highly specific genetic approach (*p21-ATTAC*) (17) accelerated fracture healing. Moreover, although previous studies demonstrated the beneficial effects on fracture healing of clearing senescent-like cells using a pharmacological approach (D + Q) (10, 11), the present study provided both a clear identification of these senescent-like cells and dissected the relative contributions of the p21 versus the p16 pathway in driving these injury-related senescent-like cells during fracture healing.

While clearance of p16-expressing senescent cells with aging prevents age-related bone loss (19, 47), it appears that the transiently senescent-like cells following bone injury are largely defined by expression of p21. These findings are entirely consistent with our previous work on focal radiation therapy (17), suggesting that p21 drives injury-specific senescence in the skeleton. Collectively, these findings lead to the hypothesis that age-related bone loss is primarily driven by p16^+^ cells, whereas acute bone loss following radiation or impaired healing following skeletal injury is principally driven by p21^+^ cells (Figure 7N). In the case of fracture, the p21^+^ senescent-like cells were principally OCH cells and a subset of neutrophils, while in the context of aging the p16^+^ senescent cells consist predominantly of osteocytes (47) and a CD24^+^ osteolineage cell population (14) (Figure 7N). We should note, however, that findings from these *ATTAC* models need to be further corroborated by complementary approaches not relying on *ATTAC*-mediated clearance of p16^+^ or p21^+^ cells. For example, studies using inducible deletion of *p16^Ink4a^* and/or *p21^Cip1^* specifically following fracture or in old mice may, in contrast to *ATTAC*-mediated clearance of senescent cells, prevent the formation of injury- or age-related senescent cells in the first place and help validate findings from the *ATTAC*-clearance models. In addition, further studies are needed to evaluate whether this dichotomy between p16 and p21 as it pertains to aging- versus injury-induced senescence is specific to the skeleton or is also true for non-skeletal tissues. Moreover, it is possible that the increase in the p16^+^ cells in the late phase of fracture healing could be relevant for late events, such as long-term stability or non-union. As such, longer-term fracture studies of 8–12 weeks need to be performed to address this issue.

As noted above, our CyTOF analyses, complemented by scRNA-seq data, also provided further insights into specific cell populations expressing features of senescence and their interactions. In particular, we found that early (days 1–3) following fracture, there was the appearance of a specific subpopulation of G5 neutrophils based on aligning our scRNA-seq data with a published mouse neutrophil atlas (33), likely contained in the Neutrophil-4 population identified by CyTOF, that is composed of mature/aged neutrophils that are highly inflammatory (33). Consistent with our findings, previous reports have identified neutrophils as early components of the fracture callus and depletion of neutrophils (e.g., using anti–Ly-6G antibody treatment) actually impairs fracture healing (50, 51). Our findings demonstrated, however, that a subset of these neutrophils begin to express features of cellular senescence, including increased p21 expression, growth arrest, and a proinflammatory SASP, along with expression of genes related to ROS pathways. Moreover, genetic reduction of these p21^+^ neutrophils, with preservation of non–p21-expressing neutrophils, led to enhanced fracture repair. Thus, specifically targeting the injury-related senescent-like neutrophils may allow the beneficial effects of non-senescent neutrophils to accelerate repair.

Our demonstration of neutrophils expressing features of senescence is not unprecedented, as Bancaro et al. (39) recently described a very similar population of neutrophils expressing senescence markers and persisting in the tumor microenvironment. In particular, these tumor-infiltrating senescent-like neutrophils also expressed p21 and were highly inflammatory, particularly enriched in SASP factors (39). Moreover, similar to our finding that clearance of senescent-like neutrophils accelerated fracture healing, these investigators also found that both genetic and pharmacological elimination of these tumor-infiltrating senescent-like neutrophils decreased tumor progression in different mouse models of prostate cancer (39). Thus, neutrophils expressing senescent-like features may be part of a broader tissue response to injury and/or cancer. It is also important to note that while terminally differentiated neutrophils are growth arrested (52), only a subset (maximum of ~20% either by CyTOF or scRNA-seq in our data) were p21^+^, but importantly, it was these p21^+^ neutrophil subsets (i.e., Neutrophil-4 by CyTOF or G5 neutrophils by scRNA-seq) that expressed senescent-like features, including high levels of SASP factors, and were reduced following AP treatment in the *p21-ATTAC* mice.

Our studies also point to important cross-talk between the G5 neutrophils and OCH cells. Previously, the Passos laboratory demonstrated that neutrophils cause telomere dysfunction in neighboring mesenchymal cells in an ROS-dependent manner (53). Moreover, senescent cells mediate the recruitment of neutrophils to their niche, potentially leading to the spread of senescence to surrounding cells (53). Thus, there appears to be a feed-forward loop between G5 neutrophils and OCH cells in the fracture callus whereby the OCH cells recruit inflammatory neutrophils (e.g., through their high expression of CXCL1, a potent chemoattractant factor for neutrophils) (25) that are high in ROS pathways, which causes DNA damage in the OCH cells, leading to a senescent phenotype, which then further recruits neutrophils. Consistent with this, our CellChat analyses (https://github.com/sqjin/CellChat)) found evidence for extensive cross-talk specifically between the G5 neutrophils and OCH cells.

In terms of the OCH cells, we were able to segregate these, using both scRNA-seq and CyTOF, into populations at multiple stages of differentiation. In contrast to the growth-arrested (Ki67^–^), p21^+^ neutrophil population that was cleared by AP treatment in the *p21-ATTAC* mice, the OCH cells were predominantly p21^+^Ki67^+^ and their abundance remained unchanged following AP treatment. Rather, these cells had a highly inflammatory phenotype, and their SASP was reduced by AP treatment concomitant with reduction of the p21^+^ neutrophils. Of note, because the CyTOF analyses were done 48 hours following the AP dose, it may be that these cells were initially cleared upon AP treatment yet appeared unchanged due to their rapid reexpansion as a result of their proliferative nature. This may explain why we observed a reduction within the growth-arrested Neutrophil-4 cluster (p21^+^Ki67^–^), but not the proliferating OCH cells (p21^+^Ki67^+^) in mice intermittently cleared of p21^+^ cells. These p21^+^Ki67^+^ cells may represent an inflammatory “pre-senescent” population at the intersection of senescence-associated growth arrest that we (14) and others (54) have recently identified both in vivo and in vitro. Clearly, the relationship of these cells to fully senescent, growth-arrested cells remains to be further clarified. In addition, further studies using lineage tracing are needed to define whether these p21^+^Ki67^+^ OCH cells originate predominantly from periosteal or endosteal cell populations (55).

The identification of *Klf4* as a key transcription factor within the detrimental OCH cells highlights a therapeutic opportunity. Recently, a small molecule was developed that inhibited the methylation of KLF4 and subsequently downregulated KLF4-mediated gene transcription (56). According to our analyses, this inhibitor might be a promising candidate for accelerating bone healing by impairing the development and/or function of these senescent cells. Specifically, further studies are needed to determine whether KLF4 expression and/or activity enables the p21^+^Ki67^+^ senescent-like OCH cell phenotype. Interestingly, KLF4 has also been identified as a key transcription factor modulating the phenotype of vascular smooth muscle cells following injury, including regulating vascular calcification (57). As such, inhibiting KLF4 expression and/or activity may have utility not just following fracture, but perhaps also following vascular injury.

In our scRNA-seq data, OCH cells expressing high levels of p21 and SASP markers were also expressing high levels of *Tgfb1* and based on CellChat, communicating via the TGF-β signaling pathway with other mesenchymal cell populations. These findings are entirely consistent with the work of Liu et al. (11) who found that senescent callus cells expressed high levels of TGF-β1 and neutralizing antibodies against TGF-β1 prevented the inhibitory effects of these senescent callus cells on the proliferation of mesenchymal stem cells. Collectively, these findings suggest that approaches to inhibit TGF-β1 signaling in the fracture callus may enhance fracture healing, and whether this would be particularly beneficial in the setting of impaired fracture healing (i.e., fracture non-unions) warrants further study. We acknowledge, however, that the effects of TGF-β1 on bone remodeling are complex, and include recruitment of mesenchymal stem cells to sites of bone remodeling (58). However, following fracture, the periosteum likely provides a pool of progenitor cells to the fracture callus (59), and the effects of TGF-β1 on inhibiting bone formation (11) may be dominant.

In order to evaluate whether our findings may extend to tissue injury beyond fracture, we further evaluated p21^+^ cell populations present following muscle injury using publically available data (12). Remarkably, muscle FAPs, which share multiple characteristics with bone marrow stromal and OCH cells (e.g., CD45^–^CD31^–^PDGFRα^+^) (38), were — like the OCH cells — the most highly enriched population for SenMayo genes (31) following muscle injury. FAPs also exhibited very similar cross-talk with neutrophils, as we found for OCH cells and neutrophils, in particular through the THBS1/CD47 pathway, which has previously been associated with the induction of paracrine senescence (34, 35). Thus, although further studies across tissues are clearly needed, our findings of a senescent-like, inflammatory mesenchymal progenitor population interacting with infiltrating neutrophils and modulating tissue repair may well extend beyond skeletal injury.

We recognize potential limitations of our work. As noted earlier, we acknowledge that findings from our *ATACC* models need to be corroborated by alternative approaches (e.g., inducible deletion of *p16^Ink4a^* and/or *p21^Cip1^* following fracture or with aging). In addition, while our studies using a genetic clearance strategy provide interventional evidence for a causal role for these injury-related senescent-like cells in impairing fracture healing, the identification of specific factors secreted by these cells that modulate fracture repair requires additional studies. As such, while our analyses provide candidate genes and pathways (e.g., the THBS1/CD47 axis from G5 neutrophils to OCH1 cells), fully addressing this issue would require extensive in vitro studies involving coculture of the relevant cell populations along with studies abrogating these pathways in vivo. Given the scope of the present work, however, we would submit that these analyses are more appropriate for future work.

In summary, our work combined a proteomic, transcriptomic, and highly specific genetic clearance approach to characterize in detail the transiently senescent-like cells that appear following tissue injury. In the case of fracture, we identified these cells as OCH cells and a specific subset of neutrophils that expressed high levels of p21 and a SASP, and we also defined important signaling pathways between these key cell populations. Moreover, by directly comparing the effects of genetic senescent cell clearance in *p21-ATTAC* versus *p16-INK-ATTAC* mice, we provided further evidence that the injury-related senescent phenotype in the setting of skeletal injury (fracture, radiation) (17) was predominantly driven by p21, particularly in the early healing phase, in contrast to age-related bone loss, which appears to be principally driven by p16 (14, 19, 47). Finally, targeting the injury-related senescent-like populations and/or the pathways we identified may prove beneficial in accelerating fracture healing and potentially repair across multiple tissues (e.g., muscle). In particular, if non-union, which occurs in up to 10% of fractures (60) and is more common in certain conditions (e.g., diabetes, chronic kidney disease, smokers; ref. 22), is shown to be associated with excess accumulation of these senescent-like cell populations, then strategies to reduce or eliminate these cells may provide a novel approach to addressing this vexing clinical problem.

## Methods

### Sex as a biological variable.

Per NIH guidelines (61), we studied both female and male mice. In order to test for possible effects of sex on our primary endpoints, we performed 2-way ANOVA tests on several important parameters of fracture healing (Supplemental Table 3) as recently recommended (62). We found that neither sex alone nor interaction between sex and time was significant, indicating that these cellular effects are not dependent on sex. Thus, both males and females were analyzed together.

### Animal studies.

All assessments were performed in a blinded fashion. Mice were housed in ventilated cages and maintained within a pathogen-free, accredited facility under a 12-hour light/dark cycle with constant temperature (23°C) and access to food and water ad libitum. We used young adult (4- to 6-month-old) C57BL/6 WT (Charles River Laboratories), *p16-INK-ATTAC* (18), *p21-ATTAC* (17) mice, and transgenic reporter mice with the p21 promoter driving GFP for our experimental procedures. Isoflurane (vaporizer, 1.5%–2% in oxygen, inhalation) was used for anesthesia during surgery for induction and maintenance until the surgery was complete. Vehicle and AP were administered intraperitoneally twice weekly. Vehicle was 4% ethanol, 10% PEG-400, and 2% Tween 80, while AP was dissolved in 4% ethanol, 10% PEG-400, and 2% Tween 80. The dose was calculated individually with 10 mg/kg body weight (BW).

Fractures for all experiments were performed as follows: Mice of comparable mean BWs received a standardized, closed diaphyseal tibial fracture. After a lateral lower leg incision, the left tibia was exposed while the tendons and muscles were protected. A transverse osteotomy with a rotary bone saw was introduced. An Insect Pin (Fine Science Tools, 26001-30, Austerlitz Insect Pin rod diameter 0.03 mm) was inserted retrogradely from the fracture to stabilize the transverse tibial shaft fracture and the distal pin end subsequently directed onto the distal fracture end. After wound closure, postoperative pain management was performed with subcutaneous BupER (0.1 mg/kg BW) and the correct position of the pin was immediately affirmed by x-ray. Normal postoperative weight bearing was allowed.

For the time course study, *n* = 23 male and *n* = 24 female C57BL/6N WT untreated mice were used (*n* = 47 in total). For the *p16-INK-ATTAC* study, 6-month-old *p16-INK-ATTAC* mice were used as described in detail elsewhere (19) (Supplemental Figure 2A). Altogether, 73 mice were used: 38 mice for vehicle treatment (22 females, 16 males) and 35 for AP treatment (20 female and 15 male mice). For the *p21-ATTAC* study, a total of 47 4-month-old mice were randomized to either vehicle (*n* = 23; 10 male and 13 female) or AP (*n* = 24; 11 male and 13 female) treatment twice weekly (Figure 2A). In addition, fluorescent dyes were subcutaneously injected intraperitoneally to trace newly formed bone: xylenol orange (0.04 mL/animal, 20 mg/mL) on day 6, calcein green (0.1 mL/animal, 2.5 mg/mL) on day 13, Alizarin red (0.1 mL/animal, 7.5 mg/mL) on day 20, and tetracycline (0.125 mL/animal, 5 mg/mL) on day 27 after fracture.

### Mouse tissue collection and assessments.

Prior to sacrifice, body mass (g) was recorded. The left tibia was stored in 0.9% saline–soaked gauze at –20°C for direct ex vivo μCT scanning (see *Skeletal imaging*) and subsequent biomechanical strength testing by standardized torsional testing (see *Torsional testing of tibiae*). In the *p21-ATTAC* and the *p16-INK-ATTAC* study, the fractured bone was embedded in methylmethacrylate and sectioned for fluorescent in situ hybridization (FISH) (see *TAF assay*). In the *p21-ATTAC* study, additional histomorphometric analyses were performed. In the CyTOF study, the fresh callus was freshly harvested at the indicated time points (see *CyTOF sample preparation*).

### CyTOF sample preparation.

Mice were sacrificed and the left tibia was isolated. Subsequently, the visually verified callus area was removed after cleaning the bone from surrounding tissue. The callus area was minced with a scalpel in FACS buffer. Pieces were then digested 3 times for 30 minutes each in 0.7 mg/mL collagenase solution (Sigma-Aldrich) at 37°C with agitation. In between steps, the solution was filtered through a wet 70-μm cell filter, washed 3 times with PBS, and the collected reaction stopped with FBS, while the remaining pieces were further digested. The samples were pooled together, resuspended in RBC lysis buffer for 5 minutes, and diluted in FACS buffer. The remaining solution was resuspended and kept on ice.

### CyTOF antibodies.

Metal-conjugated antibodies used in this study are summarized in Supplemental Table 1. Except commercially available preconjugated antibodies (Fluidigm Sciences), all antibodies were conjugated to isotopically enriched lanthanide metals using the MaxPAR antibody conjugation kit (Fluidigm Sciences), according to the manufacturer’s recommended protocol. Labeled antibodies were stored at 4°C in PBS supplemented with glycerol, 0.05% BSA, and 0.05% sodium azide. All antibodies were tested with control beads as well as positive and negative control cells. A detailed validation of the key antibodies used (e.g., p21, p16, others) is included in a recent publication from our group (14).

### CyTOF antibody staining and sample processing.

Details regarding the antibody staining and sample processing are provided in Doolittle et al. (14).

### CyTOF data analysis: initial processing and clustering.

Cleanup of cell debris, including removal of beads, dead cells, and doublets, was performed (Supplemental Figure 1A) using Cytobank software (63, 64). Visual representation of single-cell data was achieved using viSNE mapping (5,000 iterations, 100 perplexity, 0.5 theta), which is based on the *t*-distributed stochastic neighbor embedding (*t*-SNE) algorithm (65). FlowSOM clustering was performed within Cytobank (hierarchical consensus, 10 iterations) and cluster labels were assigned using established literature on skeletal cell types, with relative marker intensities per cluster visualized by heatmap. FCS files were exported, concatenated in R, and then re-uploaded for visualization of merged populations. Quantified values were exported to GraphPad Prism 8 to construct plots and perform statistical analyses. CITRUS analyses (66) were performed in Cytobank using significance analysis of microarrays (SAM) correlative association model. Nearest shrunken centroid (PAMR) and L1-penalized regression (LASSO via GLMNET) predictive association models were run simultaneously to analyze model error rates to confirm validity of the statistical model. For CITRUS assessment of median expression changes, cells were clustered by identification markers and statistics channels included all functional markers; for assessment of abundances, all markers were used for clustering. All CITRUS analyses used the following settings: 2,000 events samples per file, 2% minimum cluster size, 5 cross-validation folds, and 5% FDR.

### qRT-PCR analysis.

For callus analyses, callus and contralateral intact bone were removed as described previously (10), immediately homogenized in QIAzol Lysis Reagent (QIAGEN), and stored at –80°C. Soft tissue was removed, and a 7 mm section around the fracture site (and a 7 mm section at the same location on the intact contralateral site) was extracted and homogenized in QIAzol. Subsequent RNA extraction, cDNA synthesis, and gene expression measurements of mRNA levels by qRT-PCR were performed as described previously (67). The mouse primer sequences, designed using Primer Express software v3.0 (Applied Biosystems), are provided in Supplemental Table 2.

### Skeletal imaging: radiographical fracture healing assessment.

All imaging and analysis was performed in a blinded fashion as described by our group previously (10). In short, radiographs of the fracture site were taken under anesthesia after surgery and on a weekly basis. Therein, mice were in a supine position and both limbs extended. We assessed both the anteroposterior (ap) and lateral (lat) planes. Radiographs were evaluated by 2 blinded researchers and scored for fracture healing using the approach by Wehrle et al. (21). Quantification of the fracture callus was performed with FIJI (NIH), as described elsewhere (68).

### Skeletal imaging: ex vivo μCT imaging.

At the study endpoint, callus volume of the fracture site was evaluated. Scan settings were 55 kVp, 10.5 μm voxel size, 21.5 diameter, 145 mA, and 300 ms integration time. For the callus volume measurement, thresholds of 190 and 450 were chosen according to the manufacturer’s protocols (Scanco Medical AG).

### Torsional testing of tibiae.

Tests of torsional load were performed in a blinded fashion. The pin was removed and the tibia embedded in the tibial plateau as distal tibia. Subsequently, the torsional load was applied at speed of 5°/second for a maximum of 36 seconds. The primary endpoints were maximum rotation angle at failure (Deg) and stiffness (N-cm/degree). The maximum torque was the highest force that the bone could sustain before fracture, and stiffness was calculated from the linear portion of the loading curve (higher values for both are indicative of stronger bone) (69).

### Bone histomorphometry.

All histomorphometric analyses were performed in a blinded manner. For dynamic histomorphometry, mice were injected subcutaneously with xylenol orange (0.04 mL/animal, 20 mg/mL), calcein (0.1 mL/animal, 2.5 mg/mL), Alizarin red (0.1 mL/animal, 7.5 mg/mL), and tetracycline (0.125 mL/animal, 5 mg/mL), on days 6, 13, 20, and 27 after fracture, respectively. Details regarding bone histomorphometry analyses are as previously described from our laboratory (19).

### TAF assay.

TAF assay was performed on murine hind limbs of non-decalcified methylmethacrylate-embedded sections as previously described (13, 47).

### scRNA-seq sample preparation.

For the scRNA-seq study, we used a total of 4 mice for single-cell sequencing (2 male, 2 female) and a total of *n* = 8 (4 male, 4 female) for flow cytometry and performed surgery as described above for preparation of the samples for CyTOF. The 3 samples per bone were pooled together and resuspended in RBC lysis buffer for 5 minutes and diluted in FACS buffer. The remaining solution was resuspended and kept on ice, and sorted by FACS.

### Flow cytometry–based sorting of GFP^+^ cells.

Following the cell isolation from callus, the cells were live sorted by staining with propidium iodide (PO-PRO I, Thermo Fisher Scientific, P3581) in staining buffer. After that, the cells were electronically gated on live GFP^+^ cells based on a wavelength of 509 nm and a low expression of PO-PRO I (indicating live cells) and sorted through a FACSAria digital cytometer running FACSDiva v8.0.1 software (BD Biosciences). The GFP^+^ and GFP^–^ cells as remaining cells were either kept in QIAzol Lysis Reagent (QIAGEN) and stored at –80°C or immediately used for single-cell analysis.

### scRNA-seq analysis.

After FACS, the single-cell suspension was loaded onto the 10× Genomics Chromium device using version 2 chemistry. The samples were sequenced at GeneWiz where we targeted 50,000 reads per cell on an Illumina HiSeq 4000 device. We performed 2 lanes of sequencing using these parameters for each sample. scRNA-seq data were aligned and quantified using the 10× Genomics CellRanger Software Suite (v6.1.1) against the murine reference genome (mm10). The Seurat package (v4.1.0) (70, 71) was used to perform integrated analyses of single cells. Genes expressed in fewer than 3 cells and cells that expressed fewer than 200 genes and greater than 20% mitochondrial genes were excluded from downstream analysis in each sample. The data set was SCTransform normalized and the top 3000 highly variable genes across cells were selected. The data sets were integrated based on anchors identified between data sets before principal component analysis (PCA) was performed for linear dimensional reduction. A shared nearest neighbor (SNN) graph was constructed in order to identify clusters on the low-dimensional space (top 30 statistically significant principal components, PCs). An unbiased clustering according to the recommendations of the Seurat package was used, and a resolution of 1.4 led to 38 distinct cellular clusters. These were manually assigned to 18 cell types (Supplemental Figure 4). The heatmaps were generated in Seurat (v4.1.0) using the top 10 differentially expressed genes based on the average log_2_(fold change), after applying the FindAllMarkers function (min.pct = 0.25, logfc.threshold = 0.25). For uniform manifold approximation and projection (UMAP) dimension reduction calculations, the RunUMAP function (dims = 1:40, reduction = “pca”) was utilized, and both DimPlot (Seurat, v4.1.0) and plot_cells (monocle 3, v.1.2.0; https://cole-trapnell-lab.github.io/monocle3/) were used for plotting.

For pseudotime analyses, trajectory interference was generated via RNA velocity (72), monocle 3, and monocle 2 (https://cole-trapnell-lab.github.io/monocle-release/docs/). For RNA velocity, the raw sequencing reads from fastq files were arranged into spliced and unspliced matrices by velocyto (https://bustools.github.io/BUS_notebooks_R/velocity.html). The RNA velocity was the inferred with the stochastic model of Scvelo and Kallisto (73). Filtering out genes with no more than 20 counts in spliced and unspliced matrices reduced the subsequent Seurat object. After dimensional reduction and PCA as UMAP calculation, velocity was run onto the PCA reduction, revealing the subsequent pseudotemporal trajectory based on spliced and unspliced variants.

For monocle, an independent component analysis (ICA) dimensional reduction was performed, followed by a projection of a minimal spanning tree (MST) of the cell’s location in this reduced space. Each cell is assigned a pseudotemporal space. Monocle 2 was used to preprocess, perform UMAP reduction, and reduce the dimensionality using the DDRTree algorithm with a maximum of 4 dimensions. Subsequently, the cells were ordered and genes plotted along the reduced dimension. Differential gene testing was performed with the formula “~sm.ns(Pseudotime),” and the results were restricted by a *q* value of less than 0.1 (74, 75).

For the signaling network, CellChat (1.1.3) was utilized, aggregating a cell-cell communication network from significant signaling genes and interactions (threshold.*P* = 0.05) according to netAnalysis_signalingRole after the centrality scores were calculated in the inferred intercellular communication network (“netP”, min.cells = 10). The regulatory units analysis was performed using SCENIC (1.2.4) (76). The network analysis was conducted with Cytoscape 3.8.2 and the plugin iRegulon (77).

### Statistics.

Graphical data are shown as mean ± SEM unless otherwise specified. The sample sizes were determined based on previously conducted and published experiments, e.g., Farr et al. (19) and Saul et al. (10), in which statistically significant differences were observed among various bone parameters in response to multiple interventions in our laboratory. The used animal numbers are indicated in the figure legends; all samples presented represent biological replicates. We did not exclude mice, samples, or data points from analyses. Data were examined for normality and distribution using dot plots and histograms; all variables were examined for skewness and kurtosis. If the normality or equal variance assumptions for parametric analysis methods were not met, data were analyzed using nonparametric tests (e.g., Wilcoxon’s rank-sum test, Mann-Whitney *U* test). For parametric tests, depending on the comparison, differences between groups were analyzed by independent samples *t* test or 1-way ANOVA, where justified as appropriate. When ANOVA determined a statistically significant (*P* < 0.05) effect, pairwise multiple comparisons were performed and Tukey’s post hoc method was applied unless specified otherwise. Statistical analyses were performed using either GraphPad Prism (v9.0) or R v4.0.2 (https://www.r-project.org/). A *P* value of less than 0.05 (2-tailed) was considered statistically significant. Box-and-whisker plots show the median (line in box) and interquartile range (bounds of box), with whiskers representing minimum and maximum values.

### Study approval.

Animal studies were performed under protocols approved by the Mayo Clinic (Rochester, Minnesota, USA) Institutional Animal Care and Use Committee (IACUC).

### Data availability.

All data supporting the graphs and tables are provided in the supplemental Supporting Data Values file. The scRNA-seq data are available at the NCBI Gene Expression Omnibus repository (GEO GSE253863). The CyTOF data generated in this study have been deposited in the Mendeley database under https://doi.org/10.17632/7wzsyk6355.1

## Author contributions

DS, MLD, and SK conceived and designed research studies. DS, MLD, JLR, MNF, RLK, SJV, DGM, and MR conducted experiments and acquired data. DS, MLD, and SK analyzed and interpreted data with input and advice from NKL, AC, RJP, JFP, DGM, and JNF. DS, MLD, and SK wrote the manuscript with input and edits from all authors.

## Supplementary Material

Supplemental data

Supporting data values

## Version 1 05/16/2024

In-Press Preview

## Version 2 06/17/2024

Electronic publication
