# Supplementary material for: Osteochondroprogenitor cells and neutrophils expressing p21 and senescence markers modulate fracture repair
Source: J Clin Invest. 2024 May 16;134(12):e179834. doi: 10.1172/JCI179834 (PMC11178538; doi:10.1172/JCI179834)
Supplement: Supplemental data [file jci-134-179834-s205.pdf]

Saul et al. Supplementary Tables and Figures

SUPPLEMENTARY TABLES

Supplementary Table 1. CyTOF antibodies, conjugated metals and source.

**Timecourse Panel**			
**Marker**	**Metal**	**Vendor**	**Cat #**
CD45	089Y	Fluidigm	3089005B
Ki67	106Cd	Biolegend	103051
CD11b	110Cd	Biolegend	101201
OCN	111Cd	Santa Cruz	sc-376835
Bcl-2	112Cd	Biolegend	633502
Ly6C	113Cd	Biolegend	128039
p53	116Cd	Abcam	ab252388
Ctsk/Cathepsin K	141Pr	Abcam	ab37259
CD24	142Nd	Biolegend	101802
Dmp1	143Nd	ThermoFisher	PA5-47621
SDF-1 / CXCL12	144Nd	R&D Systems	MAB350-100
CD115 / CSF1R	146Nd	Biolegend	135521
Runx2	147Sm	Abcam	ab76956
CD140a / PDGFRα	148Nd	Biolegend	135902
CD68	149Sm	Biolegend	137002
TNFRSF11A/CD265	150Nd	BioLegend	119803
SP7	151Eu	Invitrogen	PA5-40411
CD200	152Sm	Biolegend	123802
PPARy	153Eu	Invitrogen	PA3-821A
ALPL	154Sm	R&D Systems	af2910
p16	155Gd	Abcam	ab232402
CD73	156Gd	BioLegend	127202
F4/80	158Gd	Biolegend	123101
IL-1β	159Tb	Cell Signaling	31202
CD206	160Gd	Biolegend	141702
CX3CR1	161Dy	Biolegend	149002
Sox9	163Dy	Abcam	ab220812
CD31	165Ho	Fluidigm	3165013B
Sclerostin	166Er	Abcam	ab63097
ACP5	167Er	Abcam	ab83050
Sox6	168Er	Biorbyt	orb669202
Ly-6A/Sca-1	169Tm	Fluidigm	3169015B
CD169	170Er	Fluidigm	3170018B
IL-1α	171Yb	Biolegend	503202
E11	174Yb	BioLegend	127401
CXCL1	175Lu	R&D Systems	MAB453-500
p21	176Yb	Santa Cruz	sc-6246

***p21-ATTAC* Panel**			
**Marker**	**Metal**	**Vendor**	**Cat #**
CD45	089Y	Fluidigm	3089005B
Ki67	106Cd	Biolegend	350523
CD11b	110Cd	Biolegend	101249

OCN	111Cd	Santa Cruz	sc-376835
Bcl-2	112Cd	Biolegend	633502
Ly6C	113Cd	Biolegend	128039
pATM	114Cd	Invitrogen	14-0146-82
p53	116Cd	Abcam	ab252388
Ctsk/Cathepsin K	141Pr	Abcam	ab37259
CD24	142Nd	Biolegend	101802
Dmp1	143Nd	ThermoFisher	PA5-47621
TGFB1	144Nd	Novus Biologicals	NBP2-74495
CD19	145Nd	Biolegend	115502
CD115 / CSF1R	146Nd	Biolegend	135521
Runx2	147Sm	Abcam	ab76956
CD140a / PDGFRα	148Nd	Biolegend	135902
CD68	149Sm	Biolegend	137002
TNFRSF11A/CD265	150Nd	BioLegend	119803
SP7	151Eu	Invitrogen	PA5-40411
CD200	152Sm	Biolegend	123802
PPARγ	153Eu	Invitrogen	PA3-821A
ALPL	154Sm	R&D Systems	af2910
p16	155Gd	Abcam	ab232402
F4/80	158Gd	Biolegend	123101
IL-1β	159Tb	Cell Signaling	31202
PAI-1	160Gd	Abcam	ab125687
CX3CR1	161Dy	Biolegend	149002
Sox9	163Dy	Abcam	ab220812
pSTAT1	164Dy	Novus Biologicals	AF2894-SP
CD31	165Ho	Fluidigm	3165013B
Sox6	168Er	Biorbyt	orb669202
Ly-6A/Sca-1	169Tm	Fluidigm	3169015B
Ly-6G	170Er	Biolegend	127637
IL-1α	171Yb	Biolegend	503202
CD206 (MMR)	173Yb	Biolegend	141702
SDF-1 / CXCL12	174Yb	R&D Systems	MAB350-100
CXCL1	175Lu	R&D Systems	MAB453-500
p21	176Yb	Santa Cruz	sc-6246
CD3e	196Pt	Biolegend	100302

**OCH Detailed Panel**			
**Marker**	**Metal**	**Vendor**	**Cat #**
CD45	089Y	Fluidigm	3089005B
Ki67	106Cd	Biolegend	350523
CD11b	110Cd	Biolegend	101249
OCN	111Cd	Santa Cruz	sc-376835
Bcl-2	112Cd	Biolegend	633502
Ly6C	113Cd	Biolegend	128039
pATM	114Cd	Invitrogen	14-0146-82
CD51	115In	Biolegend	153202
NFATc1 (NFAT2)	139La	Biolegend	649602
Ctsk/Cathepsin K	141Pr	Abcam	ab37259

CD24	142Nd	Biolegend	101802
Dmp1	143Nd	ThermoFisher	PA5-47621
TGFB1	144Nd	Novus Biologicals	NBP2-74495
CD19	145Nd	Biolegend	115502
LeptinR	146Nd	R&D Systems	AF497
Runx2	147Sm	Abcam	ab76956
CD140a / PDGFRα	148Nd	Biolegend	135902
SP7	151Eu	Invitrogen	PA5-40411
CD200	152Sm	Biolegend	123802
PPARy	153Eu	Invitrogen	PA3-821A
ALPL	154Sm	R&D Systems	af2910
p16	155Gd	Abcam	ab232402
Embigin	156Gd	ThermoFisher	14-5839-85
CD29	158Gd	Biolegend	102235
IL-1β	159Tb	Cell Signaling	31202
PAI-1	160Gd	Abcam	ab125687
Sox9	163Dy	Abcam	ab220812
pSTAT1	164Dy	Novus Biologicals	AF2894-SP
CD31	165Ho	Fluidigm	3165013B
BCL-XL	167Er	Abcam	ab32370
Sox6	168Er	Biorbyt	orb669202
Ly-6A/Sca-1	169Tm	Fluidigm	3169015B
Ly-6G	170Er	Biolegend	127637
IL-1α	171Yb	Biolegend	503202
Col2a1	172Yb	Abcam	ab34712
Galectin-1	173Yb	Abcam	ab225540
SDF-1 / CXCL12	174Yb	R&D Systems	MAB350-100
CXCL1	175Lu	R&D Systems	MAB453-500
p21	176Yb	Santa Cruz	sc-6246
CD3e	196Pt	Biolegend	100302
PCNA	198Pt	Biolegend	307902

Supplementary Table 2. Mouse qPCR primer sequences

Category	Gene	Forward Primer sequence (5'-3')	Reverse Primer Sequence (5'-3')
Senescence	*Cdkn2a*^{Ink4a}	GAACTCTTTCGGTCGTACCC	AGTTCGAATCTGCACCGTAGT
Senescence	*Cdkn2a*^{pan}	AGCTCTTCTGCTCAACTACGG	GGAGAAGGTAGTGGGGTCCT
Senescence	*Cdkn1a*^{Cip1}	GAACATCTCAGGGCCGAAAA	TGCGCTTGGAGTGATAGAAATC
Senescence	*Cdkn1a* ^{Var1}	AGATCCACAGCGATATCCAGACA	GTCGGACATCACCAGGATTGGAC
Senescence	*Cdkn1a* ^{Var2}	CACTTTGCCAGCAGAATAAAAGGTG	ACACTTTGCTCCTGTGCGGAAC
Senescence	*Tp53*	TCTTATCCGGGTGGAAGGAAA	GGCGAAAAGTCTGCCTGTCTT
ATTAC transgenic cassette	*FKBP-Casp8*	CAGATTATGCCTATGGTGCCACTG	GTAAACTTTGTCCAAAGTCTGTGATTCAC
Reference	*Actb*	AATCGTGCGTGACATCAAAGAG	GCCATCTCCTGCTCGAAGTC
Reference	*Gapdh*	GACCTGACCTGCCGTCTAGAAA	CCTGCTTCACCACCTTCTTGA
SASP	*Ager*	CTGGCACTTAGATGGGAAACT	TGTCTCCTGGTCTCTTCCTT
SASP	*Ccl2*	GTCTGTGCTGACCCCAAGAAG	TGGTTCCGATCCAGGTTTTTA
SASP	*Ccl3*	TCCCAGCCAGGTGTCATTTT	TTGGAGTCAGCGCAGATCTG
SASP	*Ccl5*	GCCCACGTCAAGGAGTATTTCT	ACAAACACGACTGCAAGATTGG
SASP	*Ccl7*	CCCTGGGAAGCTGTTATCTTCA	CTGATGGGCTTCAGCACAGA
SASP	*Ccl8*	CCACACAGAAGTGGGTCAGTGA	TTCAAGGCTGCAGAATTTGAGA
SASP	*Csf1*	ATTGCCAAGGAGGTGTCAGAA	GGACCTTCAGGTGTCCATTCC
SASP	*Csf2*	CCTTGAACATGACAGCCAGCTA	CACAGTCCGTTTCCGGAGTT
SASP	*Csf3*	CCTGCAGGCTCTATCGGGTAT	ATCCAGCTGAAGCAAGTCCAA
SASP	*Cxcl1*	CCGAAGTCATAGCCACACTCAA	CAAGGGAGCTTCAGGGTCAAG
SASP	*Cxcl10*	TGAATCCGGAATCTAAGACCA	TTTTTGGCTAAACGCTTTCAT
SASP	*Cxcl12*	GCCAACGTCAAGCATCTGAAA	CAGCCGTGCAACAATCTGAA
SASP	*Cxcl15*	TCCATGGGTGAAGGCTACTGT	TTCATTGCCGGTGGAAATTC

SASP	*Cxcl16*	GCACCCCTGCACATAG TCAGA	AGGACAGTGCTCCTGATGGAA
SASP	*Cxcl2*	TCAAGGGCGGTCAAAA AGTT	CAGTTAGCCTTGCCTTTGTTCA
SASP	*Fas*	CTGCACCCTGACCCA GAATAC	ACAGCCAGGAGAATCGCAGTA
SASP	*Fasl*	CCGCTCTGATCTCTGG AGTGA	CACGAAGTACAACCCAGTTTCG
SASP	*Foxo4*	CCACGAAGCAGTTCAA ATGCT	TTCAGACTCCGGCCTCATTG
SASP	*Gas6*	AGGCTCAACTACACCC GAACA	TTTAACTTCCCAGGTGGTTTCC
SASP	*Gata4*	GCTCCTACTCCAGCCC CTAC	CAGGACTGGGCTGTCGAA
SASP	*Gdf15*	TGTGCAGGCAACTCTT GAAGA	GCGATACAGGTGGGGACACT
SASP	*Hmgb1*	TCCTTCGGCCTTCTTC TTGTT	AGGATGCTCGCCTTTGATTTT
SASP	*Icam1*	GTGGCGGGAAAGTTC CTGTT	GTCCAGCCGAGGACCATACA
SASP	*Ifng*	TTGGCTTTGCAGCTCT TCCT	ATGACTGTGCCGTGGCAGTA
SASP	*Igf1*	AAAAGCAGCCCGCTCT ATCC	CTTCTGAGTCTTGGGCATGTCA
SASP	*Igfbp2*	GCCCCCTGGAACATCT CTACT	GTTGTACCGGCCATGCTTGT
SASP	*Igfbp3*	ACCTGCTCCAGGAAAC ATCAGT	TTTCCACACTCCCAGCATTG
SASP	*Igfbp4*	GCAACTTCCACCCCAA ACAGT	CCTGTCTTCCGATCCACACA
SASP	*Il10*	TGGCTCAGCACTGCTA TGCT	TGTACTGGCCCCTGCTGATC
SASP	*Il12a*	ATCCTGCTTCACGCCT TCAG	GATAGCCCATCACCCTGTTGA
SASP	*Il12b*	GCCAGTACACCTGCCA CAAAG	TGTGGAGCAGCAGATGTGAGT
SASP	*Il15*	GGCATTCATGTCTTCAT TTTGG	TCCAGTTGGCCTCTGTTTTAGG
SASP	*Il17a*	GGACTCTCCACCGCAA TGAA	GCACTGAGCTTCCCAGATCAC
SASP	*Il1a*	AAGAGACCATCCAACC CAGATC	CCTGACGAGCTTCATCAGTTTG
SASP	*Il1b*	TCAGGCAGGCAGTATC ACTCA	CACGGGAAAGACACAGGTAGC T
SASP	*Il3*	GCCTGCCTACATCTGC GAAT	CGAAAGTCATCCAGATCTCGAA
SASP	*Il4*	TCCTCACAGCAACGAA GAACAC	AAGCACCTTGGAAGCCCTACA
SASP	*Il6*	ACCACGGCCTTCCCTA CTTC	TTGGGAGTGGTATCCTCTGTGA

SASP	*Il8*	TCCATGGGTGAAGGCTACTGT	TTCATTGCCGGTGGAAATTC
SASP	*Inhba*	CAGGAAGACACTGCACTTTGA	TTCAGGAAGAGCCACACTTCT
SASP	*Irf1*	CAGCCGAGACACTAAGAGCAAA	GAGAAAGTGTCCGGGCTAACAT
SASP	*Lif*	GCTGTATCGGATGGTCGCATA	TCTGGTCCCGGGTGATATTG
SASP	*Mif*	GCCACCATGCCTATGTTCATC	GGGTGAGCTCCGACAGAAAC
SASP	*Mmp12*	GTGCCCGATGTACAGCATCTT	GGTACCGCTTCATCCATCTTG
SASP	*Mmp13*	TGAGGAAGACCTTGTGTTTGCA	GCAAGAGTCGCAGGATGGTAGT
SASP	*Mmp2*	TGTGGGTGGAAATTCAGAAGGT	ACTTGTTGCCCAGGAAAGTGA
SASP	*Mmp7*	TTGCTGCCACCCATGAATTT	TCACAGTACCGGGAACAGAAGA
SASP	*Mmp8*	TGGCTGCTCATGAATTTGGA	CATCAAGGCACCAGGATCAGT
SASP	*Mmp9*	TGAGTCCGGCAGACAATCCT	CCCTGGATCTCAGCAATAGCA
SASP	*Nfkb1*	GGCTTTGCAAACCTGGGAAT	TCCGTGCTTCCAGTGTTTCA
SASP	*Pappa*	CATCTCAGGTGTGTCGAACCA	TGCAAGGATACCAAGCATGCT
SASP	*Pdgfa*	CTCGAAGTCAGATCCACAGCAT	CAGCCCCTACGGAGTCTATCTC
SASP	*Pdgfb*	GCTGAGCTGGACTTGAACATGA	CCTCGAGATGAGCTTTCCAACT
SASP	*Serpinb2*	TTCCGCATACTGGAAACATCAG	GGATGCGTCCTCAATCTCATC
SASP	*Serpine1*	GGACACCCTCAGCATGTTCA	CGGAGAGGTGCACATCTTTCT
SASP	*Sparc*	GAGGAGGTGGTGGCTGACAA	CACCTTGCCATGTTTGCAAT
SASP	*Spp1*	GAGGAGGTGGTGGCTGACAA	CACCTTGCCATGTTTGCAAT
SASP	*Tgfb1*	AGCGCTCACTGCTCTTGTGA	GCTGATCCCGTTGATTTCCA
SASP	*Tgfb2*	GGTGGCGCTCAGTCTGTCTAC	TCTTGCGCATAAACTGATCCAT
SASP	*Tgfb3*	CTGTCCACTTGCACCACGTT	CCTAATGGCTTCCACCCTCTT
SASP	*Tgfbr1*	CGTGTGCCAAATGAAGAGGAT	AAGGTGGTGCCCTCTGAAATG
SASP	*Tnf*	GTTCTGCAAAGGGAGAGTGG	GCACCTCAGGGAAGAGTCTG
SASP	*Tnfrsf11b*	CCAAGAGCCCAGTGTTTCTT	CCAAGCCAGCCATTGTTAAT

SASP	*Tnfrsf12a*	CCGCCGGAGAGAAAAGTTTAC	GGGTGCTCCTCACTGGATCA
SASP	*Tnfsf10*	CTCTCGGAAAGGGCATTCATT	TCGATGACCAGCTCTCCATTC
SASP	*Tnfsf11*	GCTGGGACCTGCAAATAAGT	TTGCACAGAAAACATTACACCTG
SASP	*Vcam1*	GGCTCCAGACATTTACCCAGTT	CATGAGCTGGTCACCCTTGAA
SASP	*Vegfa*	GTACCTCCACCATGCCAAGTG	TGGGACTTCTGCTCTCCTTCTG

Supplementary Table 3. Assessment of sex as a biological variable. 2-way ANOVA results found no interaction effects of sex on primary endpoints of fracture healing.

Metric	Source of Variation	% of total variation	P value	P value summary	Significant?
Wehrle score	Interaction	0.1703	0.5303	ns	No
	Sex	0.1482	0.0597	ns	No
	Timepoint	91.34	<0.0001	****	Yes
Relative callus area	Interaction	0.3442	0.9887	ns	No
	Sex	0.3583	0.4390	ns	No
	Timepoint	26.03	<0.0001	****	Yes
qPCR *Cdkn1a*	Interaction	1.181	0.6265	ns	No
	Sex	0.3526	0.7895	ns	No
	AP Treatment	46.51	0.0094	**	Yes

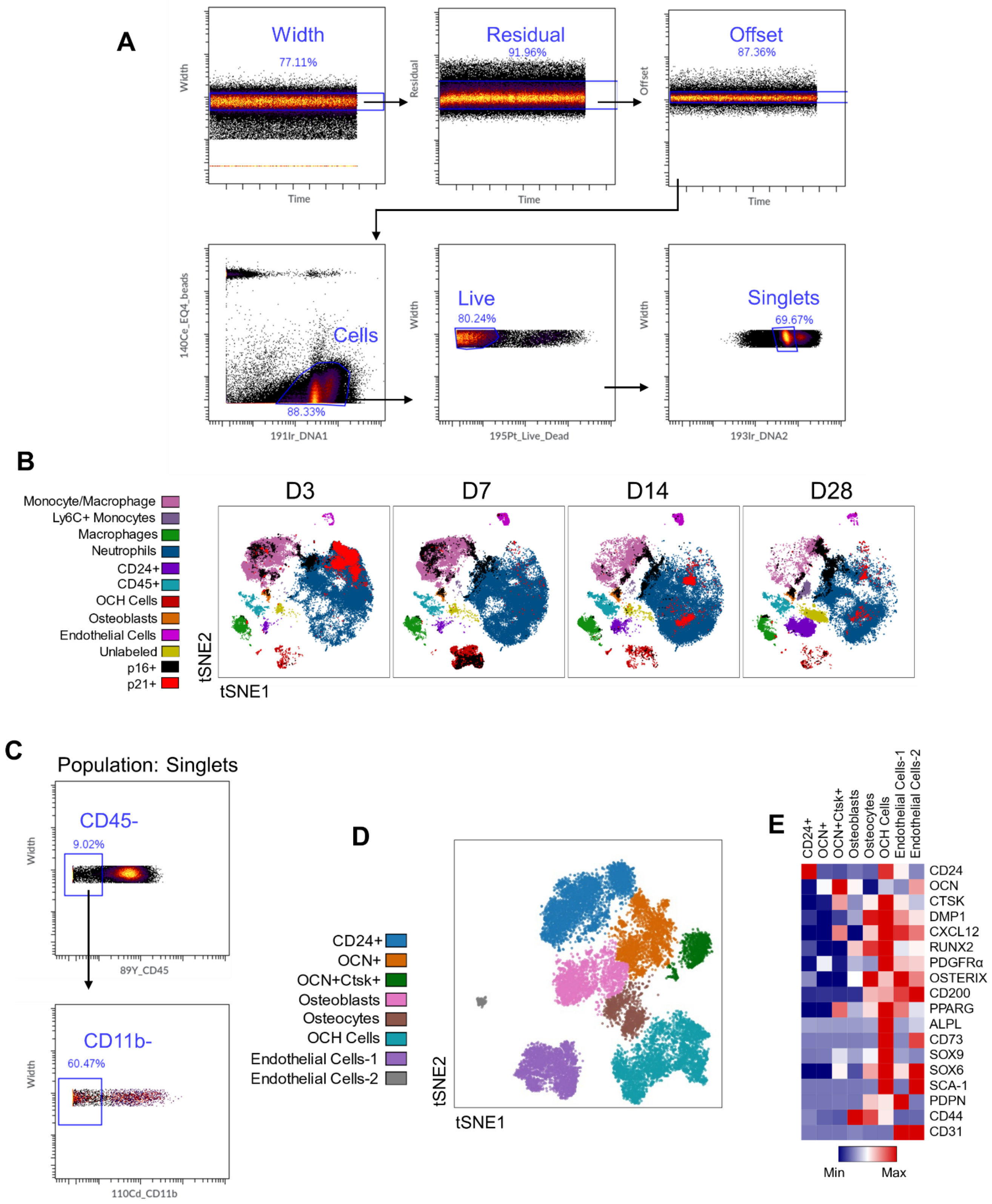

Supplementary Figure 1: CyTOF characterization of murine fracture healing. (A) CyTOF gating strategy for purification of live singlets. (B) tSNE visualization of clustered fracture callus

cell populations by CyTOF, overlaid with p16+ (black) or p21+ (red) cells. (C) Gating strategy for purification of CD45-CD11b- cells. (D) tSNE visualization of re-clustered CD45-CD11b- non-immune callus cells. (E) Heatmap of identity marker mean expression in each cluster. Day 3: n=12 mice (6 female, 6 male), day 7: n=12 mice (6 female, 6 male), day 14: n=12 mice (6 female, 6 male), day 28: n=11 mice (6 female, 5 male).

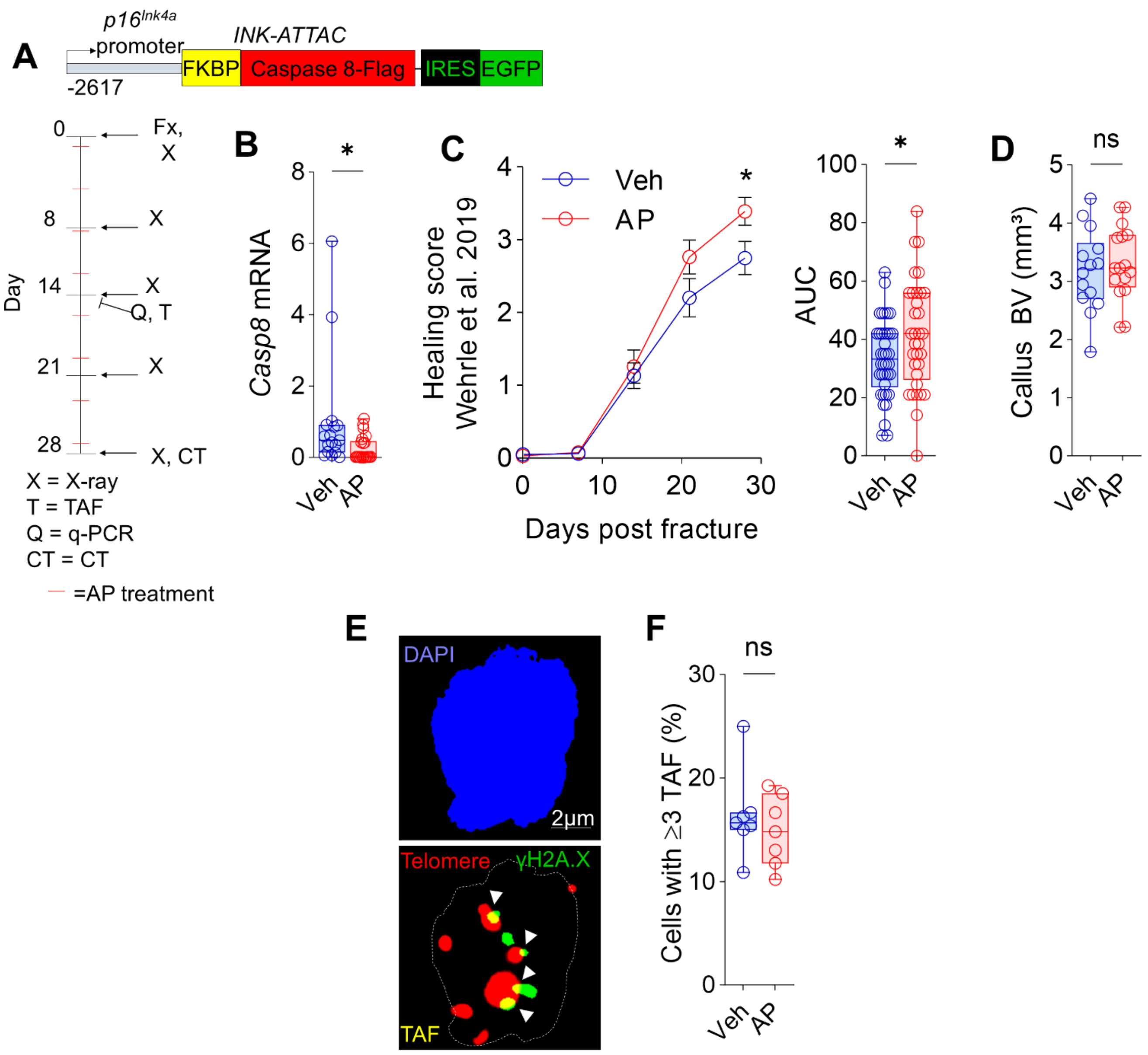

Supplementary Figure 2: p16+ cell clearance minimally affects murine fracture healing. (A) Schematic of the transgene in the *p16-INK-ATTAC* mice and overall study design. (B) qPCR measurement of INK-ATTAC transgene (*Casp8*), reduced with AP treatment (C) X-ray healing score and area under the curve (AUC) analysis in Vehicle- and AP-treated *p16-INK-ATTAC* mice over a 5-week healing period. (D) Micro-CT analysis of callus bone volume. (E) TAF (yellow, see arrowheads) were defined as sites of γH2A.X-associated DNA damage co-localized with telomeres (n=70 cells were analyzed per bone). (F) Quantification of cells with ≥3 TAFs. (B) n=17 Veh (9 female, 8 male), n=22 AP (12 female, 10 male). (C) n=40 Veh (22 female, 18 male), n=33 AP (20 female, 13 male). (D) n=14 Veh (10 female, 4 male), n=16 AP (9 female, 7 male). (E-F) n=7 Veh (5 female, 2 male), n=7 AP (5 female, 2 male). (B, D, F) Mann-Whitney or unpaired t test as appropriate. (C) Two-way ANOVA with Sidak multiple comparisons test. *$p<0.05$.

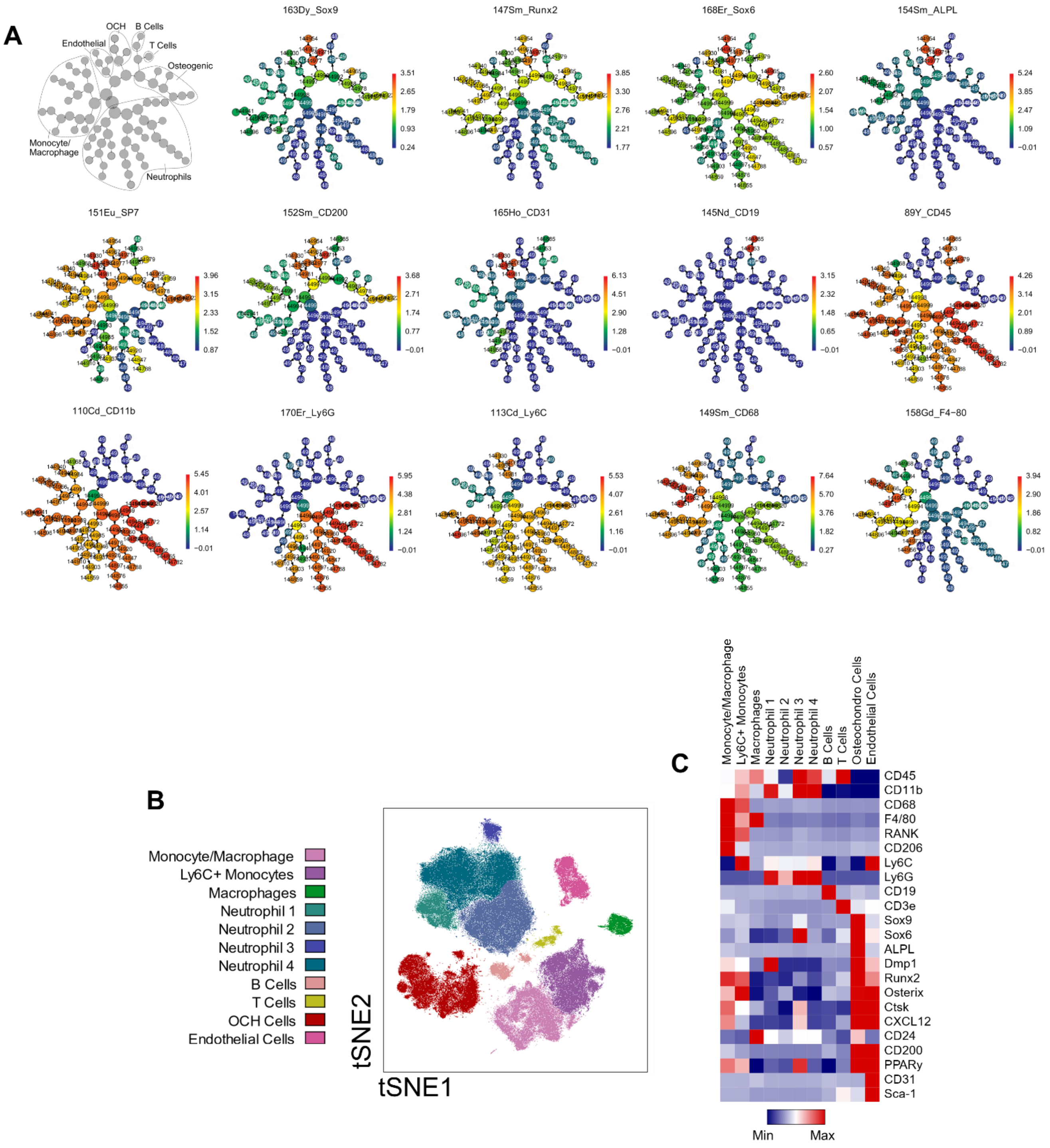

Supplementary Figure 3: CyTOF characterization of fracture callus cells in *p21-ATTAC* mice. (A) CITRUS expression plots of identity markers. (B) tSNE visualization of all clustered callus cell populations merged from vehicle- or AP-treated *p21-ATTAC* mice at D7 post-fracture. (C) Heatmap of identity marker mean expression in each cluster. n=16 Veh (8 female, 8 male), n=13 AP (7 female, 6 male).

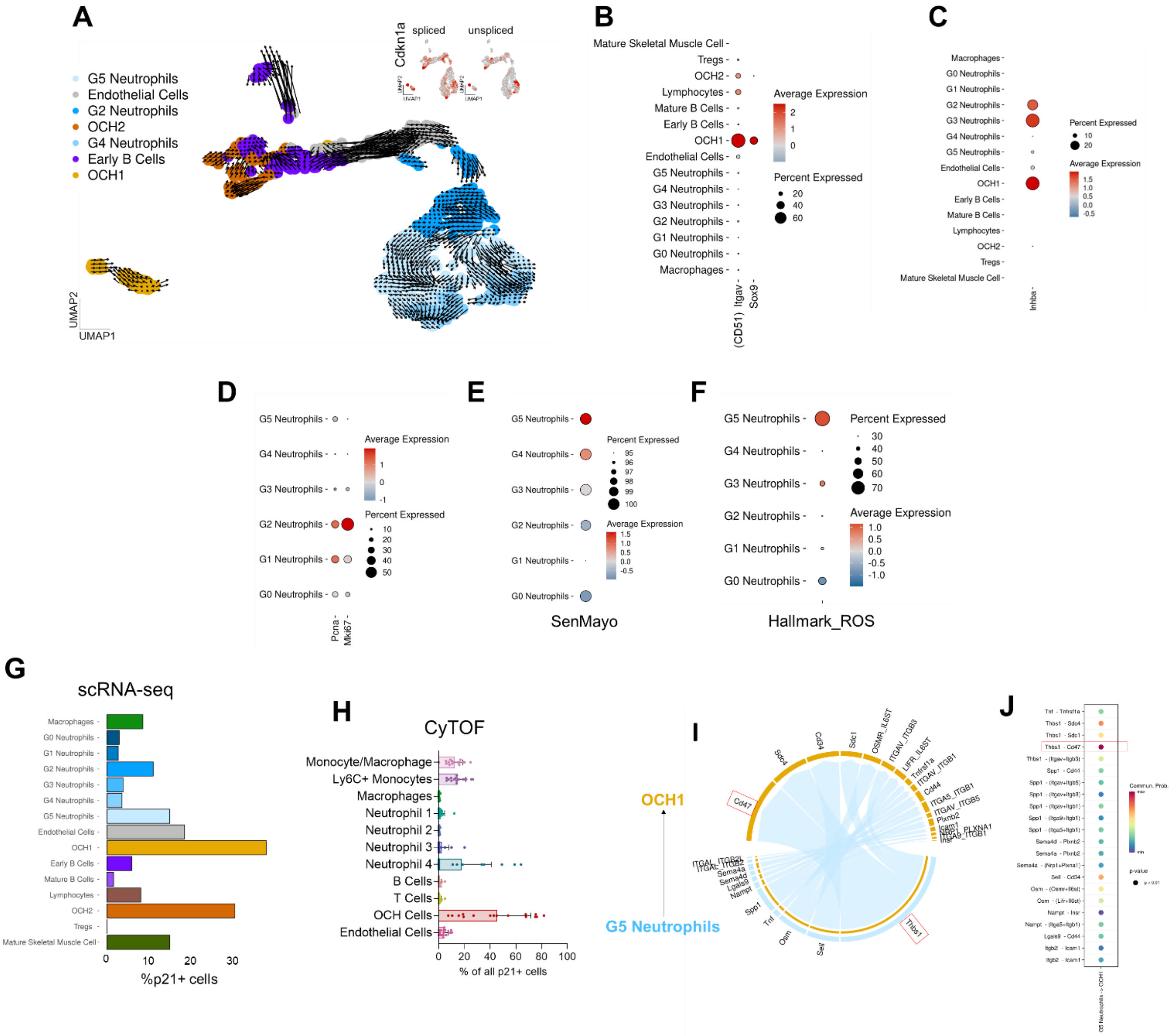

Supplementary Figure 4: Detailed analysis of callus cell scRNA-seq. (A) RNA-Velocity[68] demonstrates the detached state of OCH1 cells (lower left), while neutrophil development is gradually shown on the right developmental path. (B) OCH1 cells are highly enriched in *Itgav* and *Sox9*, while (C) high in *Inhba*, as are G2 and G2 neutrophils. (D) Proliferation markers (*Pcna* and *Mki67*) are highest in the G2 neutrophil population, but not in the G5 population. (E) The neutrophil population with the highest SenMayo score is G5, which also (F) enriches the most in the Hallmark reactive oxygen species (ROS, M5938). (G) In the scRNA-Seq dataset, the percentage of p21+ cells within the neutrophil clusters is highest in G5, which is (H) mirrored in the CyTOF dataset, where the Neutrophil 4 cells show the highest percentage of p21+ cells. (I) The communicational path preferably used by G5 neutrophils to OCH1 cells is the *Thbs1*/*Cd47* pathway, that is (J) highly enriched for these two cell types.

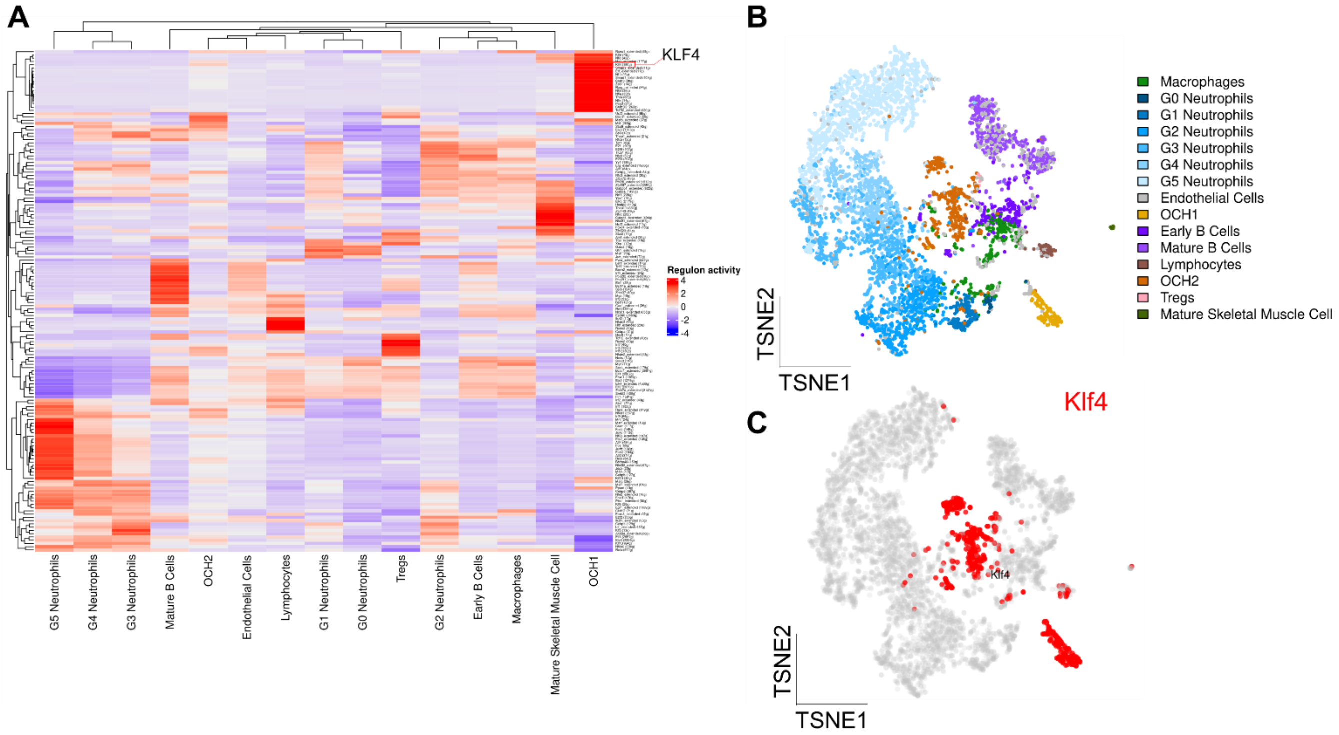

Supplementary Figure 5: SCENIC regulatory analysis of callus cell populations. (A) All significantly regulating regulons are depicted for each cell type. Notably, *Klf4* is among the key controlling genes for the OCH1 and OCH2 cells. (B) tSNE visualization after regulon assessment. (C) The *Klf4* regulon alone controls large parts of the OCH1 and OCH2 clusters.

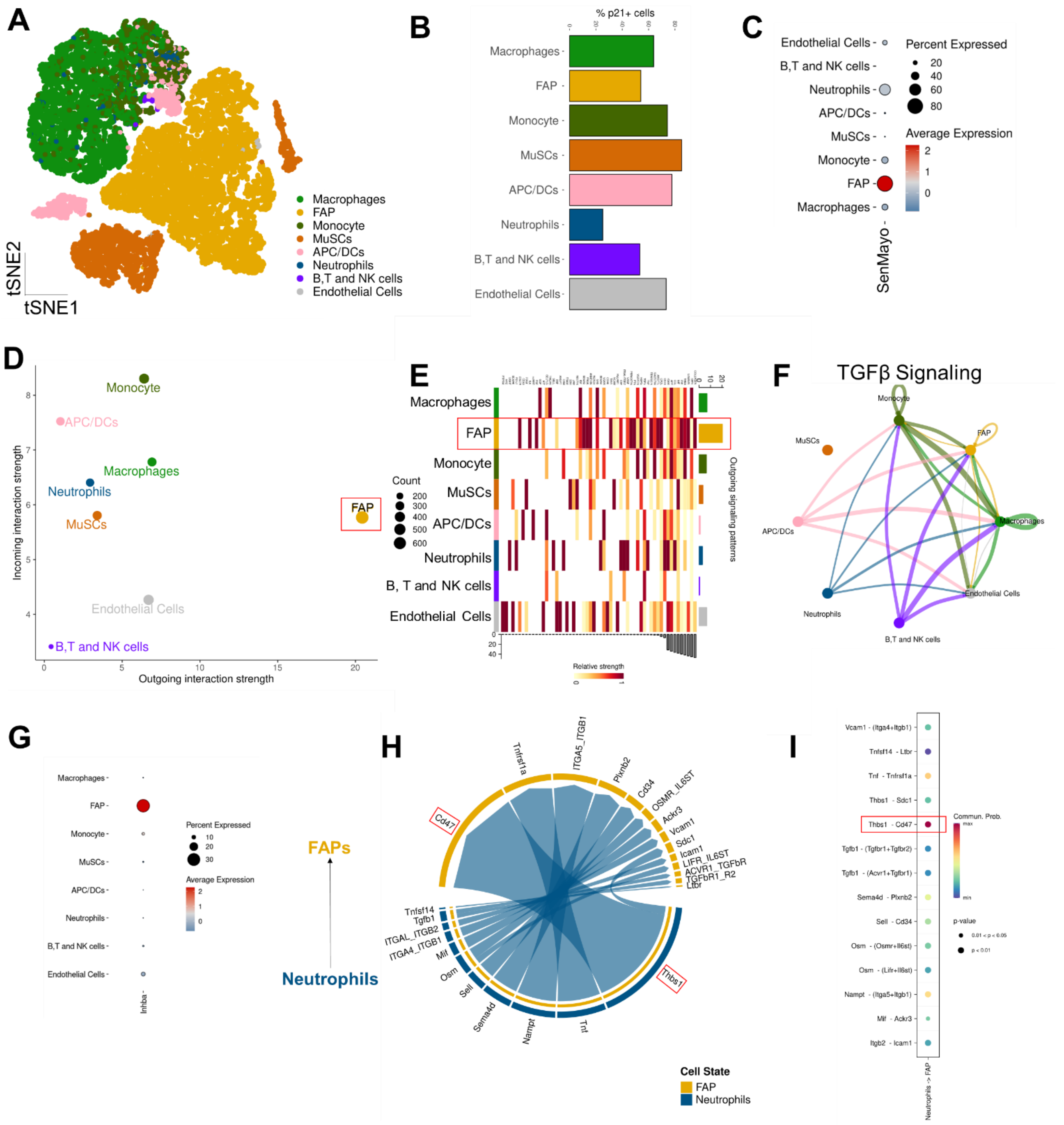

Supplementary Figure 6: Detailed analysis of skeletal muscle scRNA-seq (A) tSNE plot demonstrating the heterogeneity of murine skeletal muscle (GSE197017).[12] (B) The percentage of p21+ cells is highest in muscle stem cells (MuSCs) and antigen-presenting/dendritic cells (APC/DCs), while (C) SenMayo is mostly enriched in the fibroadipogenic progenitors (FAPs). (D, E) These FAPs are the cells with the highest outgoing interaction strength. (F) Amongst others, the *TGFβ* pathway seems to be crucial in the FAPs. (G) The FAPs are also highest in *Inhba*. (H) Similar to bone, neutrophils primarily communicate to these FAPs via the *Thbs1*/*Cd47* pathway that is (I) highly enriched for the communication neutrophils – FAPs.
